# Addressing maternal medication use during breastfeeding using clinical resources and a novel physiologically based pharmacokinetic model-derived metric: A qualitative study

**DOI:** 10.3389/fped.2023.1147566

**Published:** 2023-04-03

**Authors:** Cindy Hoi Ting Yeung, Sherilyn K. D. Houle, Philip O. Anderson, Brookie M. Best, Samuel Dubinsky, Andrea N. Edginton

**Affiliations:** ^1^ School of Pharmacy, Faculty of Science, University of Waterloo, Kitchener, ON, Canada; ^2^ Skaggs School of Pharmacy and Pharmaceutical Sciences, University of California San Diego, La Jolla, CA, United States

**Keywords:** maternal medication use, breastfeeding resources, physiologically-based pharmacokinetic (PBPK) modeling, healthcare provider interviews, thematic analysis, infant health

## Abstract

**Introduction:**

Breastfeeding has major benefits to the maternal-infant dyad and yet healthcare providers have expressed uncertainty about advocating breastfeeding when mothers are taking medications. The tendency for some providers to be more cautious in their advising approach is likely a consequence of limited, unfamiliar, and unreliable existing information on medication use during lactation. A novel risk metric termed the Upper Area Under the Curve Ratio (UAR) was developed to overcome existing resource shortcomings. However, the perception and use of the UAR in practice by providers is not known. The aim of this study was to understand existing resource use and potential UAR use in practice, their advantages and disadvantages, and areas of improvement for the UAR.

**Methods:**

Healthcare providers mainly practicing in California with experience advising on medication use during lactation were recruited. One-on-one semi-structured interviews that included questions on current practices when advising medication use during breastfeeding, and approaches to a given a scenario with and without information about the UAR were conducted. The Framework Method was applied for data analysis to construct themes and codes.

**Results:**

Twenty-eight providers representing multiple professions and disciplines were interviewed. Six main themes emerged: (1) Current Practice Approaches, (2) Advantages of Existing Resources, (3) Disadvantages of Existing Resources, (4) Advantages of the UAR, (5) Disadvantages of the UAR, and (6) Strategies to Improve the UAR. Overall, 108 codes were identified that illustrated theme topics ranging from a general lack of metric use to the realities of advising. A workflow describing current practice approaches connected all other themes. Almost all disadvantages of existing resources could be overcome by advantages of other resources and the UAR. Several improvements to the UAR were identified to address its shortcomings.

**Conclusion:**

Through interviews with providers who use resources to advise on medication use during breastfeeding, an improved understanding of current practice approaches and accessed resources was ascertained. Ultimately, it was found that the UAR would confer multiple benefits over existing resources, and improvements of the UAR were identified. Future work should focus on implementing the suggested recommendations to ensure optimal uptake of the UAR to improve advising practices.

## 1. Introduction

Breastfeeding is known to be beneficial for both the mother and infant, but its practice is often questioned during maternal medication use. According to several guidelines, only a small percentage of medications are contraindicated while breastfeeding (1–4). Yet, healthcare providers have a tendency towards advising mothers not to breastfeed during medication use (5, 6). Several factors contribute to overly cautious recommendations. First, data on medications during lactation, such as concentrations in breast milk, are limited. In fact, almost half of drugs approved in the U.S. between 2003 and 2012 (47.9%) had labels with no data on breastfeeding and only 4.7% contained human data (7). More recently, a review of 1,408 medications reported in LactMed revealed that only 2% had strong data with information in four categories (maternal drug levels, infant drug levels, effects on infants, and effects on lactation) from research studies (8). Second, even if data from studies of medications in lactation do exist and are increasing in number, disseminating this information to healthcare providers is a challenge. A cross-sectional study conducted in 2021 showed that knowledge of the new Food and Drug Administration (FDA) Pregnancy and Lactation Labeling Rule (PLLR) by pharmacists and physicians was generally low (9). Third, resources developed to improve the knowledge translation of existing drugs in lactation data have variable reliability. An evaluation of lactation recommendations of 19 medications from ten drug information resources were highly variable (10). Specifically, the number of medications recognized as low risk were different among the resources. For instance, at one extreme LactMed and Hale’s Medications and Mothers’ Milk (MMM) stated that 71% of the medications were compatible with breastfeeding, whereas at the other extreme the Physicians’ Desk Reference (PDR) cited only 5% as compatible (10).

The consequence of limited, unfamiliar, and unreliable information is reflected in healthcare providers feeling inadequately knowledgeable on maternal medication use in lactation. A review of the literature by Hussainy and Dermele (11) reported that most studies found healthcare providers to have poor knowledge and variable practices mostly guided by personal experience. These themes are further exemplified in studies by Schrempp (12), Lee (13), Long and Montouris (14), Maher and Hughes (15), and McAuley (16).

To improve healthcare provider knowledge and advising, we developed a novel risk assessment metric, the Upper Area Under the Curve Ratio (UAR). The UAR is defined by dividing the 95th percentile of simulated pediatric area under the curve (AUC) by the median adult therapeutic AUC (Supplementary S1). The simulated AUCs are produced by leveraging physiologically based pharmacokinetic (PBPK) modeling. PBPK modeling is a computational tool that uses a mathematical description of drug pharmacokinetics (PK) in the body to predict its exposure. The approach is mechanistic and “bottom up”, with physicochemical properties of the compound and system parameters (anatomy and physiology) being the two main inputs. At minimum, a daily bodyweight-normalized infant volume of milk intake model (17) and information about drug concentration in breast milk, together with the drug’s pediatric PBPK model that translates dose *via* breast milk into exposure in neonates, are needed to produce the UAR. Applying these components, we pioneered the UAR to measure relative infant exposure risk using validated lamotrigine PBPK models as a first case example in a previous publication (18).

The UAR aims to improve the reliability of current resources and address the sparse data that exist on drugs in lactation. Current metrics do not account for important considerations when predicting breastfed infant risk to maternal medications. These factors are the anatomy and physiology of the infant, age-dependent factors (e.g., milk intake volumes and elimination rate), and variability in the infant and maternal populations. The UAR adequately addresses these components, for instance, by incorporating variability to capture breastfed infants who may be at most risk of high drug exposure from mothers with a pharmacogenotype resulting in the excretion of potentially dangerous levels of drug in milk. Further, the UAR does not depend on data that are typically unavailable, such as breastfed infant plasma drug concentrations. Thus, the UAR can be calculated for drugs where only sparse data are available. If data are available (e.g., a few infant plasma drug concentrations), they are used only for confirmatory rather than exploratory purposes. Increasing work that validates pediatric PBPK models to accurately predict breastfeeding infant exposures gives confidence in our workflow and UAR determination (18). With more drugs assessed with our workflow, eventually we can rank the drugs according to their potential risk and focus resources on those with significant risk (i.e., highest UAR).

Although the UAR was created in an effort to improve available clinical resources, how it is perceived and potentially used in practice by healthcare providers has not been formally assessed. To further understand healthcare provider perspectives, it is important to gather information on how resources are currently being used, whether there is a need for the UAR in addition to current resources, how the UAR could be used in current practice, whether the UAR would confer benefits, which healthcare providers would particularly benefit from use of the UAR, and how the UAR could be further improved for clinical practice [e.g., approaches to disseminate the UAR beyond its publication in Yeung (18)]. Thus, the objective for this study was to understand existing resource use and UAR use in practice, their advantages and disadvantages, and areas of improvement for the UAR through one-on-one semi-structured interviews with healthcare providers. We hypothesized that the novel risk metric will confer multiple benefits over existing resources, and improvements in the metric and how it is described to healthcare providers will be ascertained.

## 2. Methods

### 2.1. Sampling and recruitment

Stratified purposeful sampling was employed to recruit healthcare providers from a variety of backgrounds (teratogen/lactation information specialists, nurses, pharmacists, and physicians) and multiple disciplines (midwifery, neonatology, obstetrics, pediatrics, and lactation consultants). To ensure we attained perspectives from a range of experiences, we also specifically recruited from settings where providers may have less exposure to this type of advising, including emergency medicine and community pharmacies. Healthcare providers who were eligible to participate must have met the following criteria: able to communicate in English, experienced in providing or advising care for mothers taking medications while breastfeeding, and familiarity with drugs and breastfeeding clinical resources used to advise clinicians or patients. Breastfeeding clinical resources included both informational online or book resources (LactMed, Hale’s MMM, Briggs’ Drugs in Pregnancy and Lactation, etc.) and metrics (Relative Infant Dose (RID), Milk-to-Plasma (M/P) ratio, Hale’s Lactation Risk Categories (L1-5), etc.).

Recruitment was conducted through several strategies. Emails were sent to mailing lists and website listings of University of California San Diego (UC San Diego) Faculty from School of Medicine Departments of Family Medicine and Public Health; Obstetrics, Gynecology, and Reproductive Sciences (Nurse Midwifery Program); Pediatrics (Divisions of Gastroenterology, Hepatology, and Nutrition; and Neonatology), and Skaggs School of Pharmacy and Pharmaceutical Sciences (Division of Clinical Pharmacy; and Affiliate Faculty Community Pharmacists). Lactation and teratogen services, hospital perinatal units, and hospital neonatal intensive care units (NICUs) staff were also contacted through mailing lists. Snowball sampling and personal connections were also used to enhance recruitment. Recruitment was primarily performed in San Diego, California because of the high breastfeeding rates and to present perspectives with some similarities to attain saturation. Saturation occurred when no new information appeared to emerge during data analysis.

Individuals interested in participating contacted the study coordinator, provided consent, and scheduled an interview. Written informed consent was obtained from all participants prior to their interview. Participants received a US $50 gift card for appreciation of their time and possible travel costs.

This study received ethics clearance through the UC San Diego Institutional Review Board (IRB #803063) and the University of Waterloo Research Ethics Board (REB #43702). NVivo software (QSR International Pty Ltd., released in March 2020) was used for qualitative data analysis.

### 2.2. Data collection

Participant demographics on gender identity, race/ethnicity, practicing discipline, primary occupation and specialty, and measures of experience providing or advising care for patients breastfeeding or considering breastfeeding were attained through a written questionnaire. Measures of experience included number of years of experience, International Board of Lactation Consultation Examiners certification, Academy of Breastfeeding Medicine membership, and frequency of inquiries about medication use during breastfeeding from patients and other providers.

Semi-structured interviews of 25–60 min were conducted between June and September 2022 by the study coordinator (PhD candidate who developed the UAR, with a life sciences, research methods, and pharmacometrics background) either in-person or through video call. The interview guide received feedback from healthcare providers within the study team and colleagues. The final version of the guide is presented in Supplementary S2 and included questions to generate discussion on the provider’s current practices when advising on medication use in breastfeeding and, given a scenario, how they would proceed in practice currently and with information about the UAR metric. Provided materials on the scenario, and the introduction to and application of the UAR are shown in Supplementary S1. Interviews were audio recorded and subsequently transcribed.

### 2.3. Data analysis

The Framework Method, as described by Gale (19), was applied as the overarching analysis method to guide the thematic analysis of textual data. This method is commonly used to create a new structure for summarizing textual data to answering research questions. Briefly, descriptive or conceptual labels were assigned to excerpts of the interview transcripts and referred to as “codes”. Two members of the study team (CHTY and SD) independently coded the interview transcripts. Applied codes were compared and reviewed and disagreements were discussed and resolved. After coding the first few transcripts, an agreed set of codes to apply to all subsequent transcripts, also known as an analytical framework, was developed and presented in a code book.

To assess the extent of agreement between the coders, inter-rater agreement determined from Cohen’s Kappa statistic was calculated using a coding comparison query. Interviews with a Kappa statistic less than 80% were reviewed, and coding strategies and descriptions were clarified. To analyze the codes and identify themes that grouped the codes by similarities and interrelated ideas or concepts, data were charted into a framework matrix. The framework matrix provided a summary table depicting the codes as columns and participant quotations as rows to visualize themes and patterns. Illustrative quotations were selected to represent the resulting themes and codes.

## 3. Results

### 3.1. Participant demographics

Twenty-eight participants were interviewed and their demographics are presented in Table 1. Of the participants, five had International Board of Lactation Consultation Examiners certification and one had Academy of Breastfeeding Medicine membership.

**Table 1:** Study participant characteristics.

Characteristic^a^	Number of individuals
**Gender identity**	
Man	4
Non-binary	0
Woman	24
**Race/ethnicity**	
Aboriginal/American Indian/Alaska Native	0
Asian	7
Asian-White	1
Black or African American	0
Hispanic, Latino, or Spanish origin	0
Middle Eastern or North African	2
Native Hawaiian or Other Pacific Islander	0
White	18
**Primary practice setting and role**	
Midwifery	
Nurse Midwife	3
Neonatology	
Neonatologist	2
Pharmacist	2
Registered Nurse	1
Obstetrics	
Obstetrician	1
Pharmacist	1
Registered Nurse	1
Pediatrics	
Nurse Practitioner	1
Pediatrician	3
Pharmacist	1
Teratogen/Lactation Information Specialist^b^	4
Adult Critical Care	
Pharmacist	1
Community Pharmacy	
Pharmacist	4
Emergency Medicine	
Pharmacist	1
Family Medicine	
Physician	2
**Experience providing or advising care for lactating breastfeeding**	
<1 year	0
1–3 years	2
4–6 years	2
>6 years	24
**Frequency of patient or other healthcare provider inquiries on medication risk while breastfeeding**	
Daily	4
Weekly	10
Monthly	12
Less than Monthly	2

^a^ For demographics questions regarding identity, participants had the options of selecting “prefer not to disclose” and “prefer not to say”.

^b^ Participants’ primary role was defined as Teratogen/Lactation Information Specialist and their healthcare provider roles included Genetic Counsellors, Nurse Practitioners, and Registered Nurses.

### 3.2. Themes and codes

**Figure 1:**
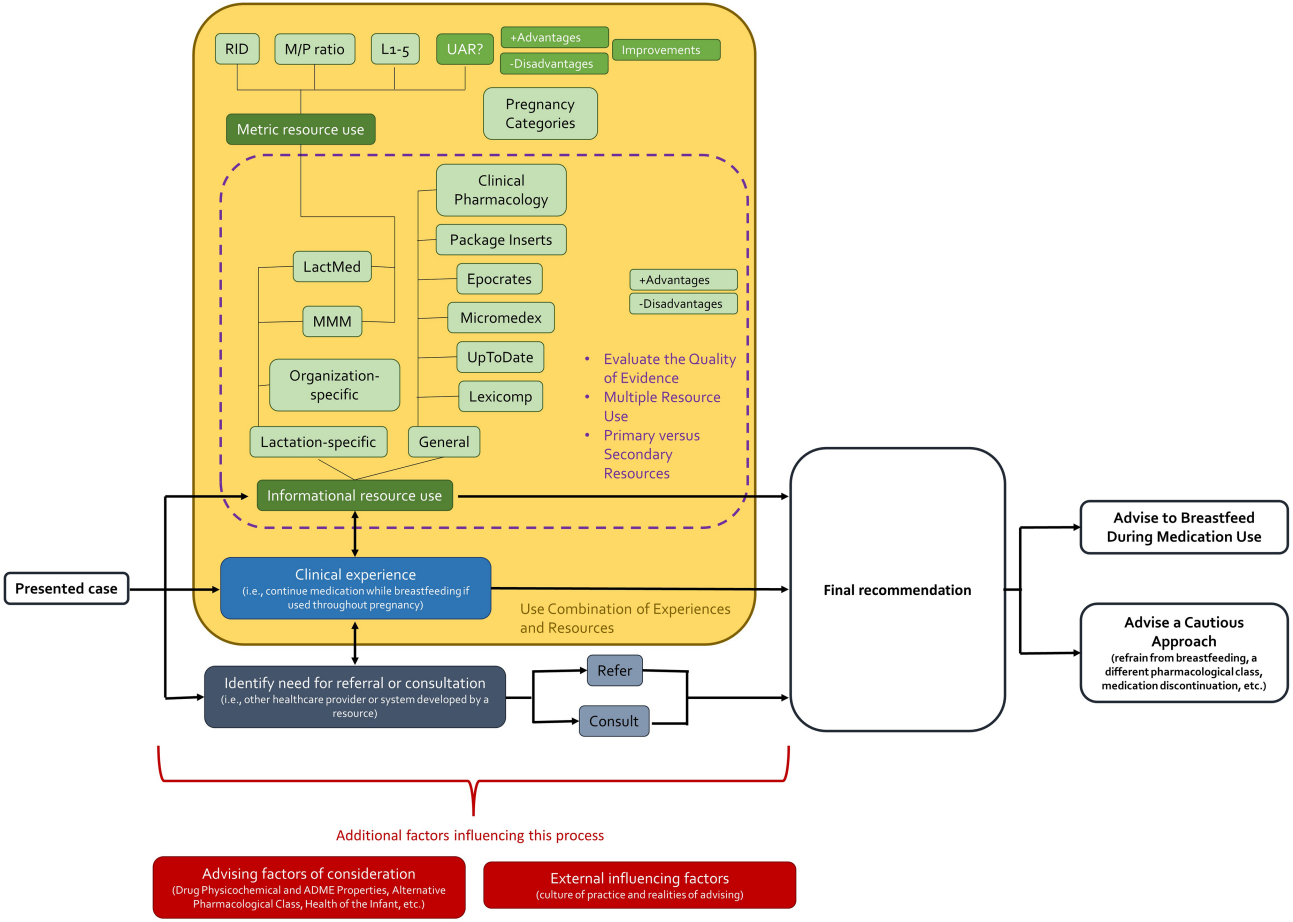
A flow diagram of summarized healthcare provider current practice approaches when advising mothers taking medication during breastfeeding. RID, relative infant dose; M/P ratio, milk-to-plasma ratio; L1-5, Hale’s Lactation Risk Categories, 1–5; UAR, upper area under the curve ratio; MMM, Medications and Mothers’ Milk; ADME, absorption, distribution, metabolism, and excretion.

The Framework Method produced several themes and codes. Six broad themes emerged: (1) Current Practice Approaches, (2) Advantages of Existing Resources, (3) Disadvantages of Existing Resources, (4) Advantages of the UAR, (5) Disadvantages of the UAR, and (6) Strategies to Improve the UAR. Figure 1 depicts Current Practice Approach as the connecting theme to all others by outlining an opportunity when the UAR could be applied in practice, a reflection of the disadvantages of existing resources and how they could be addressed by advantages of existing resources and the UAR (Table 2), and the disadvantages of the UAR with strategies for improvement (Table 3). For each theme, their representative codes are defined in Supplementary S3 and their selected illustrative quotations are shown in Supplementary S4. In the following sections, overarching themes and their descriptive codes will be presented. Themes and codes will be labelled with T# and C#, respectively. Participants who contributed to each code will be referred to by their study identifier, BFR# (Supplementary S3).

**Table 2:** Codes describing disadvantages of existing resources with potential to be addressed by advantages of existing resources and the UAR.

Disadvantages of existing resources	Advantages of existing resources and the UAR
• Areas of Subjectivity [C67]	Existing resources: • Evidence to Support Use [C4] • Trusted Authors [C10] • Summarizes and References Evidence [C8] UAR: • Numerical Metric [C20] • Objective [C21]
• Non-average Cases Not Considered [C74] • Co-medications Not Considered [C68] • Effect on Milk Not Considered [C70] • Infant Age Not Considered [C72] • Maternal Dose Not Considered [C73]	Existing resources: • Distinguishes and Provides Various Types of Data [C3] UAR: • Addresses Clearance Differences [C12] • ddresses Multiple Considerations [C14] • Addresses the Worst Case Scenario [C18] • Addresses the Age of the Infant [C16]
• Inaccessible [C71]	Existing resources: • Accessible Through the Institution [C1] • Generally Accessible [C6] • Patient-friendly [C7] UAR: • Visual Representation [C24] • Can Share with Other Providers and Patients [C19]
• Unclear Conclusions [C80]	Existing resources: • Summary Statements [C9]
• Easily Outdated [C69]	Existing resources: • Up to Date [C11]
• Overreliance on Case Reports and Published Data [C76]	UAR: • Addresses Scarcity of Published Information [C15]
• Too Broad [C78]	Existing resources: • Comprehensive [C2]

**Table 3:** Codes describing the disadvantages of the UAR and strategies for improvement.

Disadvantages of the UAR	Strategies to improve the UAR
• Co-medications Not Apparent [C81] • *In utero* Exposure Not Apparent [C83] • Metabolites Not Apparent [C86] • Multiple Administrations to Mother Not Apparent [C87] • Prematurity Not Apparent [C93]	• Separate by Specific Cases and Scenarios [C108]
• Potential to Appear Subjective or Misinterpreted [C92]	• Explain More About How the Model was Made (Inputs and Assessments) [C97] • Explain More About UAR Advantages [C98]
• Difficult to Understand or Too Complex [C82]	• Provide Guidance to Interpret the UAR [C106] • Make Visual Representation Essential [C102] • User Friendly for Non-pharmacists [C100] • User Friendly for Pharmacists [C101] • Provide a Definitive Bottom Line [C104]
• Lack of Maternal Perspective [C84]	• Provide a Greater Maternal Emphasis [C105]
• Limited Information on Adverse Effects (Exposure-Response Relationship) [C85]	• Provide Prospective Predictive Evidence [C107]
• Not Enough for Clinical Decision-making [C88]	• Combine the UAR with Another Resource [C96]
• Unusable in Current Form (Too Novel) [C94]	• Add a Summary Statement [C95] • Give Specific Training [C99] • Overcome Simulation Skepticism [C103]

#### 3.2.1. Current practice approaches

Current Practice Approaches [T3] encompassed healthcare provider use of resources that are considered when addressing medication use in breastfeeding, and how these resources are applied given a scenario (Supplementary S1). Providers described a workflow that they typically employed when presented with a case (Figure 1).

##### 3.2.1.1. Three main approaches: informational resource use, clinical experience, and identify need for referral or consultation

When provided with a scenario of a mother who has epilepsy and is taking lamotrigine, providers gravitated towards one of three initial actions: to use informational resources [Resource as a First Go-to; C58], clinical experience, or involve additional sources of expertise.

Application of clinical experience mainly focused on advising the patient to Continue Medication as a First Go-to [C28], with knowledge that lamotrigine was taken during pregnancy and thus breastfeeding resulting in less exposure than *in utero*. The immediate recommendation to continue the medication appeared to be based on clinical experience. For example, knowledge that discontinuing anti-seizure drugs taken during pregnancy is generally not advised, thus breastfeeding while on the medication may be most reasonable (BFR03). As another example of using experience, a provider expressed that Continue Medication as a First Go-to [C28] is highly relevant for healthy term infants and thus safety during pregnancy should reflect safety during breastfeeding (BFR15).

Alternatively, providers responded to the presented case with identified need for referral [Refer to Other Provider; C56] or consultation [Reliance on Other Provider or Resource; C57]. Referrals described a preference of the provider to pass decision-making in patient advising to another provider. The referral was often in the form of sharing some information to the patient paired with advising them to contact their primary care physician (BFR20) or directly contacting the patient’s provider for their recommendation or decision (BFR06, BFR27, and BFR28). Among all providers, the act of referring was rare, but were more likely to be practiced by community pharmacists. These participants tended to highlight the realities of advising, including a lack of awareness of and access to lactation-specific resources [Inaccessible; C71] (BFR06, BFR11, and BFR20) which can be due to hurdles in their institution to attain such materials [Institution Needs Resource Justification; C51] (BFR06 and BFR20) and patient information, such as electronic medical records for diagnosis codes [Lack of Information About the Patients; C52] (BFR06 and BFR16) to perform more informed advising.

As another form of identify need for referral or consultation use, healthcare providers would rely on the assessment or advice of another provider (NICU pharmacist, lactation consultant, etc.) or from a resource (LactMed summary statement, Hale’s L1-5, etc.). In contrast to referrals, these consultations were not made for the necessity of decision-making nor judgments due to the realities of advising for some healthcare providers. Consultations were mainly to lactation consultants and health system pharmacists such as those specializing in the NICU or pediatrics. Participants voiced their appreciation for consulting providers with experience and training (e.g., knowledge in pharmacokinetics and interpreting metrics) in advising maternal medication use during breastfeeding (BFR01, BFR02, BFR03, BFR14, BFR18, and BFR28). An example of consulting a resource with reliance on a metric or appraisal conducted by the author, providers would cite a high level of dependency on Hale’s L1-5 categories as a quick method of assessment of risk (BFR25 and BFR27). One provider noted not delving deeper into the information on, nor Hale’s risk assessment of, the medication if it was categorized as L5 (BFR25).

The majority of healthcare providers practiced Resource Use as a First Go-to [C58]. Accessed informational resources were separated into lactation-specific or general. Lactation-specific resources included LactMed, Hale’s MMM, and organizational-specific resources such as MotherToBaby information sheets commonly accessed by their teratogen specialists. General resources consisted typically of pregnancy and lactation sections of UpToDate, Lexicomp, Clinical Pharmacology, and package inserts. Regardless of which informational resources were employed, providers applied three practices: Evaluate the Quality of Evidence [C31], Multiple Resource Use [C46], and Primary vs. Secondary Resources [C48]. In reviewing the existing published studies provided by their accessed resources, providers commonly made assessments on the quality of available evidence. Quality assessments consisted of considering the study designs (case report, case study, extensive PK study, etc.), study population (age of infant, maternal dose received, etc.), and dose to response data availability and type (drug levels measured in milk and infant plasma, reported adverse effects in infant, etc.). Nearly all participants cited Multiple Resource Use [C46] and many used Primary vs. Secondary Resources [C48]. This practice consisted of referring to a tertiary resource that was typically their first go-to and frequently accessed, while other informational resources were examined afterwards or only as needed. Some providers noted using general informational resources as primary resources since they provided concise background information and later accessed lactation-specific resources such as LactMed or MMM for more depth (BFR04). Conversely, other providers accessed lactation-specific resources first for full information, followed by a general resource if they found the former to be insufficient for the medication (BFR017).

Beyond informational resources, healthcare providers would access metric resources often reported in lactation-specific resources (LactMed and MMM). Metric resources included the RID, M/P ratio, Hale’s L1-5, and FDA Pregnancy Categories. A little over half of providers reported a Lack of Existing Metric Use [C45]. Many of these providers expressed being unfamiliar with metrics due to a lack of exposure to them during their education and training. Nearly all physicians across disciplines lacked metric use and preferred to rely on their team’s dedicated pharmacist to account for the metrics because of their training. Almost half of the interviewed health system pharmacists cited a dearth of metric use, with one provider explaining that their application did not align with their practice approach. In referencing the RID, the health system pharmacist mentions not using the RID threshold and instead focusing on each maternal-infant pair’s uniqueness and overall risk vs. benefit (BFR05). The remaining health system pharmacists who did apply the RID in practice applied the metric in specific scenarios, including a Mother on Co-Medications [C63], a Mother with Conditions [C64], Comparing within Drug Class [C61], to Explain a Range of Outcomes in Infants [C62], for a New Medication [C65], and for Reassurance Along with Other Resources [C66].

While the FDA Pregnancy Categories were not intended as an example of a lactation metric resource, interviews revealed that this system was considered in current practice [Pregnancy Categories (Using or Avoiding Them); C47]. Community pharmacists tended to apply the Pregnancy Categories, which are available through general information resources such as Clinical Pharmacology. For example, one community pharmacist noted that a medication classified as category C would prompt physician referral (BFR06). In contrast to this approach, another provider expressed trying to avoid using these reproductive categories since they were intended for pregnancy and deemed inadequate for use in both pregnant and lactating populations (BFR05).

Once a healthcare provider had taken a first go-to approach, it was common practice to employ one of the other two strategies thereafter (Figure 1). More often, resource use and clinical experience were applied together and thus coded as, Use Combination of Experiences and Resources [C59]. Several providers stated using the RID as a metric resource and screening tool, but also applied the metric in context with other information and clinical experience such as how much actually gets into breast milk, options to try other medications, knowledge of the baby exposed to the medication *in utero*, and variation of exposures across infants.

##### 3.2.1.2. Considered factors in advising

Several factors were considered by healthcare providers along the advising process when presented with the case scenario (Figure 1). These factors included components related to the drug, infant, mother, breastfeed, and the provider’s general advising approach. The factors were not applied in any specific order during the advising process, nor were they prescribed to a single approach (e.g., only when informational resource use took place). For instance, some providers discussed considering maternal health early on their advising (BFR07 and BFR12), while other providers may acknowledge this factor later in their process.

For drug-related factors, providers would seek an understanding of Drug Physicochemical and Absorption, Distribution, Metabolism, and Excretion (ADME) Properties [C33]. For example, needing to be aware of the medication’s absorption properties such as steroids not passing well into breast milk especially *via* nasal administration (BFR07). Factors related to the infant and mother include their health [Health of the Infant; C35 and Health of the Mother; C36], whether there was Drug Use in Pregnancy [C34], Information on Drug Used in Infants [C37], Maternal Co-medications [C38], Maternal Dose Taken [C39], and Alternative Pharmacological Class [C32]. Breastfeeding factors were Type of Breastfeeding (Exclusive or Partial) [C44] and Time of Breastfeed Relative to Dose [C43].

Neonatologists and health system pharmacists in neonatology, and teratogen/lactation information specialists were more likely to consider the entirety of the mentioned factors. For example, one neonatologist reflected on safe maternal medication alternatives, lowered risk of medication to infant *via* breast milk compared to pregnancy, preterm vs. term status of the infant (i.e., thoroughly explaining the benefits of breast milk since preterm parents tend to be more cautious), the condition of the mother and need for the medication, and possibility to discard pumped milk to get the medication out of the maternal system for a breastfeed (BFR15).

Additionally, healthcare provider general advising approaches were factors to account for in their current practice methods. Four such factors were examining Risks and Benefits (Analysis) [C40], a Team Approach (Present or Absent) [C42], Select Drug Cases for Non-Resource and Resource Use [C41], and Approach for Lack of Evidence [C27]. The majority of providers performed a Risks and Benefits (Analysis) [C40] as part of their advising by making a thoughtful assessment to weigh the risks and benefits of breastfeeding during medication use. The Team Approach (Present or Absent) [C42] described whether healthcare providers experienced multiple providers in the patient’s care being involved in the advising process. A far greater number of providers stated a presence rather than an absence of a team approach.

It was evident that some healthcare providers had Select Drug Cases for Non-Resource and Resource Use [C41]. Within disciplines, common medications were prescribed, and thus clinical experience and knowledge were applied rather than seeking additional resources. Providers would discuss distinct situations in which informational resources were and were not needed. An example of all three general advising approaches in practice comes from an emergency department health system pharmacist (BFR09). The provider mentioned being asked by other emergency providers (physicians and nurses) for consultation on a newly started patient medication that caused the emergency department visit (i.e., an adverse drug reaction). These medications were typically pain medications and antibiotics and the provider felt that their current approach to advising breastfeeding while on these drugs determined from prior use of informational resources and clinical experience, was sufficient. In contrast, if a less familiar medication was introduced, such as an antidepressant, the provider would use informational resources in the advising process. Throughout the advising, the provider described accounting for the risks of an untreated condition (i.e., mother not taking their medication) to both the mother and infant (e.g., can affect infant development if mother has depressive symptoms).

Lastly, when certain drugs did not have enough information in existing resources, some providers had a specific Approach for Lack of Evidence [C27]. Providers tended to cite a manual search for studies through the internet, and use of metrics including the M/P ratio and Hale’s L1-5 when there is not a lot of evidence available (BFR02, BFR07, and BFR10).

##### 3.2.1.3. General outcomes and external influencing factors

Following healthcare provider descriptions of their Current Practice Approaches [T3] to address the case scenario, a recommendation was made that broadly followed two categories: (1) Advise to Breastfeed During Medication Use [C26] and (2) Advise a Cautious Approach [C25] (Figure 1). For the former approach, providers would work towards having the infant breastfeed rather than defaulting to a simpler recommendation to not breastfeed. Providers who practiced this approach tended to reflect on multiple advising factors, practiced either Continue Medication as First Go-to [C28] or Resource as First Go-to [C58], and exemplified Use a Combination of Experiences and Resources [C59]. On the other hand, the latter approach led to recommendations such as refraining from breastfeeding, using a different pharmacological class, and discontinuing medication. Providers would suggest alternatives such as pumping and dumping, withholding breast milk until a later infant age, and any indication of potential exposure to infants would lead them to be more cautious (BFR06, BFR11, and BFR12).

To arrive at these two main recommendation pathways, external factors could influence decision-making (Figure 1). Culture of practice acted as an external impact where providers would acknowledge the Pro-breastfeeding Culture of California [C30], which provided an abundance of breastfeeding supports (lactation consultants, teratogen information specialists, breast milk donor banks, etc.) and the benefits of breastfeeding were widely known and advertised (BFR02, BFR06, BFR15, and BFR24). Conversely, a Culture of Leaning Towards Caution [C29] was noted to be prevalent. Healthcare providers would remark that other providers advise not to breastfeed even though the medication is known not to enter breast milk, over-recommend pumping and dumping, and have a lack of awareness that most medications are compatible with breastfeeding (BFR02, BFR05, BFR08, and BFR27). The interviewed providers cited adult primary care providers as mainly adopting this culture of advising. Much of this perspective could be due to the realities of advising. Several realities were faced by providers that could influence advising, including Concern for Liability [C49], Concerns Relaying Evidence-based Decisions [C50], Institution Needs Resource Justification [C51], Lack of Information About the Patients [C52], Minimal Time for Clinical Decision Making [C53], Variable Patient Health Literacy [C55], and Motives of Manufacturers [C54].

#### 3.2.2. Disadvantages of existing resources

Disadvantages of Existing Resources [T4] outline healthcare provider perceived drawbacks to currently used resources to address medication use while breastfeeding. These disadvantages were encountered during informational and metric resource use (Figure 1) and could lead providers to be selective in their use of materials. Many of the identified shortcomings of existing resources can be addressed by the Advantages of Existing Resources [T1] and Advantages of the UAR [T2] (Table 2) discussed in section 3.2.3 of this paper.

##### 3.2.2.1. Areas of subjectivity

Several healthcare providers thought that existing resources had Areas of Subjectivity [C67] (Table 2). Many of the comments on this disadvantage were universal across resources, in recognition that searching on medication use during breastfeeding can lead to a plethora of results with many of the resource authors providing their opinions that can be based on a selected study to create their own conclusions (BFR09). One provider voiced the disadvantage of making quick decisions based on another individual’s evaluation, specifically, authors and developers of informational and metric resources (BFR12). This method of resource use may lead to less critical thinking in clinical practice. A resource metric thought to be subjective was described as being “soft”, not applied evenly, based on small study sample sizes, opinionated, and potentially adversely impacting drug policies (BFR02 and BFR09).

##### 3.2.2.2. Several factors not considered

Healthcare providers cited numerous factors that are important for advising that are not considered in most current resources. These factors were Non-average Cases Not Considered [C74], Co-medications Not Considered [C68], Effect on Milk Not Considered [C70], Infant Age Not Considered [C72], and Maternal Dose Not Considered [C73] (Table 2). First, for non-average cases were not considered, providers noted the lack of information on the upper and lower percentiles of exposed breastfeeding infants to maternal medications (BFR06). One neonatologist elaborated on the paucity of data in preterms with unique considerations such as different renal clearances, neurodevelopmental stages, and bodyweights from reported term infants (BFR15). Second, co-medications are not addressed by existing resources. Providers felt it was not possible with current resources to assess the risk of multiple medications a hypothetical mother would be taking, and on supplements containing multiple ingredients (BFR01 and BFR17). Third, one provider described a resource lacking information on drug effect on milk supply which can influence advising practices (BFR01). Fourth, providers commented the lack of infant age taken into consideration (BFR15 and BFR16). Fifth, providers noted that resources generally do not include the doses and specific drug formulations breastfeeding mothers used in studies (BFR07 and BFR28).

##### 3.2.2.3. Inaccessible

Inaccessible [C71] resources was described as a disadvantage by several of healthcare providers (Table 2). Frequently, providers pointed out resources that needed to be purchased, and in some cases, only a physical copy format was available. An accessibility example with an online resource, such as LactMed, includes the idea that high literacy levels would be needed for families to understand the material (BFR01). Additionally, some medications were difficult to find in current resources, especially when other countries and jurisdictions use alternative drug names (BFR23).

##### 3.2.2.4. Unclear conclusions

Although not a common concern, some healthcare providers did note that some resources had Unclear Conclusions [C80] due to lack of summary statements which would be useful in making recommendations to their patients (BFR04 and BFR25) (Table 2).

##### 3.2.2.5. Easily outdated

Many healthcare providers identified that existing resources were Easily Outdated [C69] (Table 2). Most providers referenced physical resources that were not up to date since they required at least a year to produce a revised publication. It was noted that an annual update was not enough to keep up with rapidly changing information on drugs in lactation risk. Online resources were not exempt from concerns of outdatedness. Providers gave examples of drugs that they had inquired about but could not be found in online resources (BFR07 and BFR18).

##### 3.2.2.6. Overreliance on case reports and published data

Several healthcare providers recognized the universal problem of existing resources solely relying on scarce published data on drugs in lactation (Table 2). The data are typically in the form of case reports and studies with small sample sizes thereby resulting in limited certainty in study conclusions and generalizability to their patients. Providers noticed the impact of Overreliance on Case Reports and Published Data [C76], especially when recommendations are forced to conclude that there is insufficient information to advise for or against medication use during breastfeeding (BFR14 and BFR16). Because of insufficient published data, providers were aware that often the adverse effects of a drug to a breastfed infant through maternal medication use are unknown (BFR05, BFR22, and BFR24). Without informing mothers on expected drug side effects to the breastfed infant, monitoring for effects of concern and general risk-benefit analyses become difficult to conduct.

##### 3.2.2.7. Too broad

Multiple healthcare providers have classified existing resources as Too Broad [C78] (Table 2). Providers specifically identified general resources as having broad and limited information as compared to lactation-specific resources (BFR05). The lack of more detailed information, such as bioavailability and drug clearance in an infant, was also recognized as missing in current resources (BFR15).

##### 3.2.2.8. Overreliance on a single resource

Healthcare providers highlighted the negative consequences of relying too heavily upon specific resources (Table 2). For example, providers explained the impact of package inserts and the PDR which typically specify that the medication should not be taken while breastfeeding, thus at times unnecessarily leading patients to be overly cautious (BFR01, BFR03, BFR10, and BFR27). Another example of Overreliance of a Single Resource [C75], was with a provider noting that a metric resource intended to be a screening tool is commonly being used for definitive decision-making, thereby bypassing a proper risk-benefit analysis (BFR03). Furthermore, the RID was frequently overgeneralized by other providers applying the arbitrarily proposed 10% cut-off definitively. One provider explained that although a drug has an RID >10%, the drug is not necessarily high risk to the breastfeeding infant, especially when the medication has been directly administered to pediatric populations (BFR07). As an additional example, one provider explained that certain benzodiazepines having a low RID may mislead providers into thinking that the medication is a low risk to the infant when that is not always the case (BFR10).

##### 3.2.2.9. Perceived lack of reported information due to resource

In contrast to Overreliance on Case Reports and Published Data [C76] as an underlying disadvantage among all existing resources, Perceived Lack of Reported Information Due to a Resource [C77] describes current resources that tend to not include available published evidence (Table 2). Providers observed that some informational resources would state there were not enough studies when in fact studies exist in the literature (BFR16). Moreover, supplements, bioactives, new medications, and medications to treat rare conditions were thought to be missing from existing resources (BFR17 and BFR18).

##### 3.2.2.10. Too much information or text-heavy

Healthcare providers identified a disadvantage in informational resources that were labelled as Too Much Information or Text-heavy [C79] (Table 2). A provider explained that listing study after study and going through their summaries could get one lost in the content (BFR03). Particularly, for emergency department health system pharmacists, going through each study could be anxiety-inducing and suboptimal for making quick decisions with high-risk patients (BFR09).

#### 3.2.3. Advantages of existing resources and the UAR

Advantages of Existing Resources [T1] and Advantages of the UAR [T2] outline healthcare provider perceived benefits of currently used resources and the novel UAR metric, respectively. These advantages were considered at the informational and metric resource use stage (Figure 1). Similar to Disadvantages of Existing Resources [T4], the advantages could persuade some providers to use some existing materials over others. In this section, areas where the Disadvantages of Existing Resources [T4] have potential to be addressed by the advantages of existing resources and the UAR are outlined (Table 2). Current resource disadvantages of Overreliance of a Single Resource [C75], Perceived Lack of Reported Information Due to a Resource [C77], and Too Much Information or Text-heavy [C79], were unable to be addressed by existing resources nor the UAR. The remainder of this section consists of Advantages of Existing Resources [T1] [Familiarity; C5] and Advantages of the UAR [T2] [Addresses Exposures (AUC); C13, Addresses the Maternal-infant Pair; C17, Opens Up the Thought Process; C22, and Understand Existing Observations, Evidence, and Recommendations; C23] that do not necessarily combat Disadvantages of Existing Resources but could be seen as an added value to the current advising landscape.

##### 3.2.3.1. Strategies to reduce areas of subjectivity

Several advantages of resources considered to be less subjective were discussed. Strategies that these resources employ include Evidence to Support Use [C4], Trusted Authors [C10], and Summarizes and References Evidence [C8] (Table 2). For Evidence to Support Use [C4], one healthcare provider noted an improvement of a general informational resource over the years where there was a published study showing its developments over the past few decades (BFR03). Trusted Authors [C10] was a key advantage for most providers, especially when they were aware of the authors’ academic and practice background (BFR01, BFR04, and BFR25). In referencing the improvement of a general informational resource over the years, a provider commended the addition of editors with appropriate skillsets in the lactation population (BFR03). Experience with resources and trusting the authors’ process also created a perception of Trusted Authors [C10]. For example, providers would confirm that a resource author had gone through all available studies and that the presented evidence was accurate (BFR17, BFR22, and BFR26).

To further reduce the potential for subjective resources, the UAR was considered an advantage by serving as an Objective [C21] and a Numerical Metric [C20] (Table 2). Providers recognized the strength of having a numerical objective metric free from author personal interpretations of existing study data (BFR04). Additionally, the idea that the UAR is derived from data and not from subjective interpretation was thought to be a positive (BFR10). One provider recognized that because the UAR is developed from data, its results are reproducible and thus Objective [C21] (BFR06). The concept of a Numerical Metric [C20] was thought to give a more concise judgement for medication use while breastfeeding compared with existing resources that were vague and left to provider interpretation (BFR08). Some providers preferred the numeric format of the UAR which was easier to interpret and could be easily added to their existing resources (BFR09 and BFR12). One neonatologist explained that a numerical metric would especially be useful in the NICU since providers tend to be number-focused (BFR14).

##### 3.2.3.2. Ability to consider several factors

Healthcare providers valued that most informational resources Distinguishes and Provides Various Types of Data [C3] to address issues from other resources neglecting non-average cases, co-medications, effect on milk, infant age, and maternal dose (Table 2). The fact that resources divide their information by study types (animal vs. human studies and case reports vs. large clinical trials), maternal components (measured breast milk and plasma drug concentrations), infant components (measured plasma drug concentrations, adverse reactions, and whether the medication has been used in pediatrics), and potential alternative medications was helpful to consider different aspects of existing evidence (BFR05, BFR07, BFR09, BFR10, BFR12, BFR14, BFR21, BFR25, BFR27, and BFR28). An advantage to these existing resources is that information about missing factors such as effect on milk could easily be added to the existing categorized framework.

More concretely, the UAR already offers opportunities to overcome the typically neglected factors as the novel metric Addresses Clearance Differences [C12], Addresses the Worst Case Scenario [C18], Addresses the Age of the Infant [C16], and Addresses Multiple Considerations [C14] (Table 2).

Clearance differences were considered in the UAR, with one neonatologist impressed that renal clearance was accounted for, especially in the NICU setting (BFR15). Another provider found that the ability of the UAR to be used in different metabolizer statuses was an asset (BFR21).

Using the UAR to identify the worst case scenario rather than the average case was thought to be valuable. One pediatric health systems pharmacist described the UAR as being able to provide the worst case scenario because the comparison is with the 95th percentile exposure in infants compared to the median exposure in adults (BFR03). Another provider recognized the importance of the UAR in a scenario where existing resources may deem a medication to be mostly low risk, but the UAR would be able to demonstrate a point of risk (BFR09). In line with another provider’s observation, identifying a point of risk would be an advantage of the UAR to show which drugs might be of higher risk in terms of outliers (BFR19).

Many providers recognized the significance of the UAR to account for infant ages. It was helpful to understand that risk of drug exposure to infant varies across ages and could dictate periods of time for the presence or absence of caution. Providers explained the specific value to their advising in understanding risk from early infant ages (i.e., exposures peaking at the first 2 weeks of life) when infants are most vulnerable and in cases of highly lipophilic drugs, receiving high fat colostrum (BFR24 and BFR27).

Finally, the UAR has the ability to address multiple considerations. Providers would reference the study material depiction comparing the UAR with existing metrics (Supplementary S1**)** and appreciated that the UAR could address multiple factors at once (BFR01, BFR06, BFR09, BFR10, and BFR22). One teratogen/lactation information specialist understood that the UAR considered vulnerable children, metabolites, systemic exposure, pediatric concerns, and development of the gastrointestinal tract as a function of age (BFR10). Providers also noted that the ability to address multiple considerations would deem the UAR to be more individualized and specific to the situation rather than a one-size-fits-all approach (BFR13, BFR21, and BFR26). Again, neonatal perspectives reflected on the utility of the UAR to account for multiple considerations in the NICU where age, renal clearance, protein binding levels, bioavailability, maternal pharmacogenotypes, and other factors are particularly influential to preterm exposures (BFR15 and BFR26).

##### 3.2.3.3. Improved accessibility

Healthcare providers identified several current resources that were Generally Accessible [C6] and Accessible Through the Institution [C1] to overcome accessibility shortcomings of physical copy and paid resources (Table 2). General accessibility of resources was a common advantage expressed by providers. Providers identified informational resources as Generally Accessible [C6] when they were readily available at any electronic device, convenient to access without needing extra steps to view the resource, free-of-charge, and simple to read (e.g., summary table of ADME and physicochemical properties). Resources were also found to be accessible through the provider’s institution. In inquiring about the resources providers used in current practice, it appeared that the frequently accessed general informational resources were those available through their institution. One provider noted this observation by stating their preference to a general resource first because it is readily available at their institution (BFR03). For community pharmacists, there was a strong tendency for Use of Package Inserts [C60] and Clinical Pharmacology due to their work settings and organizational subscriptions (BFR6, BFR11, and BFR16). Teratology/lactation information specialists were able to access their own unique institutional databases entered by other specialists in their institution (BFR10).

Accessibility to existing resources also benefit from being Patient-friendly [C7] (Table 2). One provider described an informational resource as quick to access and in plain English to print out for patients for knowledge empowerment and improved decision-making (BFR06). Similarly, the UAR was thought to be a metric resource that providers Can Share with Other Providers and Patients [C19] (Table 2). For instance, a neonatal health systems pharmacist explained that it would be useful to share the UAR with the patient’s neonatologist and primary care physician, especially for unusual medications since the UAR provides more information (BFR12).

To further improve accessibility, the UAR offers Visual Representation [C24] of potential exposure risk to the breastfeeding infant *via* maternal medications (Table 2). Multiple providers described the benefit of having graphical and concise representations (i.e., exposure across age groups boxplots and exposure table) which helps to visually interpret data and show patients in their advising process.

##### 3.2.3.4. Summary statements for clearer conclusions

Providers found that informational resources such as LactMed included short, quick, and useful Summary Statements [C9] (Table 2). These statements were thought to pull all available data together and synthesize a clear and concise recommendation based on the information (BFR02). One provider described the advantage of acknowledging all published information, regardless of strong or weak evidence, and providing a consensus on risk with breastfeeding with alternatives (BFR09).

##### 3.2.3.5. Up to date to overcome outdatedness

The faster updates of online resources as compared to physical published copies was acknowledged as an advantage of existing resources [Up to Date; C11] (Table 2). Providers commended resources such as LactMed that provide a monthly update with an exact timestamp of the update (BFR02).

##### 3.2.3.6. Avoids overreliance on case studies and published data

Only one healthcare provider noted that the UAR Addresses Scarcity of Published Information [C15], thereby removing the necessity to rely on case studies and published data (Table 2). The provider mentioned the UAR being more data-driven without relying on single case study reported results, and that there is evidence to support the risk estimate it produces (BFR18).

##### 3.2.3.7. Comprehensive to overcome reporting too broadly

Healthcare providers identified resources such as LactMed, as a Comprehensive [C2] resource that has considered the entirety of available information (BFR04 and BFR17) (Table 2). When providers were satisfied with their Comprehensive [C2] resource, they tended to forego using further resources for their advising (BFR07, BFR12, and BFR21). Other resources such as Reprotox were considered Comprehensive [C2] in describing agents which can be particularly helpful when the product is a less known herbal (BFR07).

##### 3.2.3.8. Familiarity

Although the high level of Familiarity [C5] of existing resources would not necessarily overcome a specific disadvantage of current resources, it was brought up frequently by providers as an advantage. When sharing a recommended course of action to other providers, one provider explained bypassing a buy-in by using a well-known and accepted resource in their institution (BFR03). Other providers noted their inclination to use certain general informational resources because they are familiar with them overall (i.e., in their daily care of patients) (BFR04 and BFR22). Additionally, when resources became familiar, they were deemed easy to access and simple to use (BFR09, BFR17, and BFR26).

##### 3.2.3.9. Addresses exposures (AUC)

A unique benefit of the UAR is that it Addresses Exposures (AUC) [C13]. Providers recognized the ability of the UAR to provide exposure assumptions as an improvement over current dose-based metric resources such as the RID (BFR03 and BFR04). One neonatologist summarized the strength of providing exposure estimates by explaining that the UAR provided information from the predicted dose in milk, to the bioavailability to the infant, to the infant’s clearance ability from the bloodstream, and how long the medication remains in the infant (BFR15). One pediatrician noted the UAR going beyond the M/P ratio and RID by incorporating the entire process from the dose administered to the mother, how much gets into breast milk, how much the infant gets exposed to, and the infant’s biology (BFR02). Providers also used the UAR to frame their advising in terms of level of exposure. For example, by reviewing the UAR for a drug, the provider could make a quick observation that the medication results in a tiny exposure and thus is not too concerning to the maternal-infant pair (BFR22).

In using exposures to define risk, one provider described calculating an infant’s theoretical PK as less subjective for decision-making (BFR14). Another provider described how they would use the exposure estimates by giving an example of infants potentially reaching adult therapeutic levels and having elevated transaminases (BFR15).

##### 3.2.3.10. Addresses the maternal-infant pair

Having a metric resource that Addresses the Maternal-infant Pair [C17] was seen as an advantage to several healthcare providers. Providers found the UAR to be beneficial in performing a relative comparison with mother and infant exposures (BFR04 and BFR05). Especially when viewing the predicted adult and infant exposure boxplots across age groups, one provider appeared to account for the maternal-infant pair by voicing a thought process that reassured to continue the medication and to breastfeed during the first week of life and be more vigilant after 2 months of postnatal age (BFR09). Another provider perceived the UAR to be advantageous for considering the maternal-infant pair more broadly, which would benefit neurologists to simultaneously account for the mother and infant (BFR17). Another provider identified a further way the UAR Addresses the Maternal-infant Pair [C17] by concluding that risk to the infant based on maternal exposures would most likely be accurate (BFR25).

##### 3.2.3.11. Opens up the thought process

Healthcare providers often demonstrated a detailed thought process initiated after being introduced to the UAR and how it could be used in practice. The UAR Opens Up the Thought Process [C22] by prompting providers to consider factors that they may have not considered with existing resources. Mainly, providers went beyond dose considerations and reflected more deeply in the components and implications of current metric resources as referenced in Supplementary S1. A health system pharmacist specializing in neonatology described how providers might see a low RID and consider the medication to be low risk to the infant, however, seeing the UAR might prompt retrieving cord blood levels in the first few days postpartum, a deeper thought into whether the infant is truly at the 95th percentile, and developing strategies for a monitoring plan (BFR03). In a similar comment, a registered nurse in obstetrics described moving from the RID for a yes or no type of answer, to the UAR which forces considerations on the age of the infant, dose, exposure boxplots across ages, and exposure percentiles to aid in counselling (BFR13).

Another provider commented on how each presented case is individualized because the UAR guides providers to consider factors they may have not accounted for (BFR07). For most providers, seeing the exposures and UAR metric across infant age groups helped reflect on level of caution throughout breastfeeding (BFR12, BFR17, BFR22, and BFR23). In visualizing the lamotrigine exposure histograms across different age groups, one provider explained that the plots might prompt providers to see potential risk and encourage the patient to speak with their neurologist if there is sub-optimal seizure control, and on the other hand to remain on the medication if seizure control has been attained (BFR12).

##### 3.2.3.12. Understand existing observations, evidence, and recommendations

In addressing the case scenario, the UAR helped to Understand Existing Observations, Evidence, and Recommendations [C23]. Looking at infant exposure predictions across age groups allowed one provider to reflect on an observation that infants tend to have a small portion of their glomeruli at birth and yet the UAR demonstrated it is possible to have lower risk at early age as compared to later age because other factors were at play (BFR02). Another provider introduced a way they might use the UAR, which would be to see whether predicted exposure levels would match their observations in clinical practice (BFR03). For other providers, seeing the UAR for lamotrigine was reassuring as it reinforced their expectations from clinical experience (BFR02, BFR03, BFR05, and BFR09). For instance, an emergency department health systems pharmacist explained the UAR providing reassurance to continue breastfeeding especially during the first week of life and identify areas of potential high exposures later in life (BFR09).

#### 3.2.4. UAR disadvantages and strategies for improvement

Table 3 presents codes describing the Disadvantages of the UAR [T5] matched with identified Strategies to Improve the UAR [T6] that have potential to overcome current shortcomings. This section starts with codes describing the path to understanding the UAR which were captured when healthcare providers were first introduced to the novel metric and asked questions or commented on the UAR to develop their understanding. Information from this code identified where the UAR could improve to better describe the metric to providers. The remaining codes described in this section outline Disadvantages of the UAR [T5] and Strategies to Improve the UAR [T6] deliberately discussed by the participants. In Figure 1, disadvantages and areas for improvement are presented alongside its advantages as they would likely be considered altogether in deciding resource use during the advising process.

##### 3.2.4.1. Path to understanding the UAR

The path to understanding the UAR involved inquiries and comments about exposure comparisons between adults and infants, and between infants across age groups [Exposure Comparisons; C89]; how to interpret the exposure estimates [Interpreting the Exposure Estimates; C90]; and how to interpret the UAR [Interpreting the UAR; C91].

In studying the relative exposure estimates between adult and infant from the provided lamotrigine case scenario, healthcare providers voiced their interpretations. Some providers viewed minimal crossover in the boxplots of adult and infant lamotrigine exposures, with infants only receiving miniscule exposures compared to adult (BFR01 and BFR28). Other providers also recognized the potentially low risk to infants due to minimal exposure overlap, but acknowledged that some infants above the 95th percentile could reach adult levels (BFR15).

There were several inquiries on interpreting the exposures, in the form of AUC_0−∞_, from the illustrated histograms and boxplots. Providers asked for assistance to interpret the histogram y- and x-axes and whether milk or plasma concentrations were shown, how the infant AUC_0−∞_ was derived, whether the PBPK model used to produce the simulated infant AUC_0−∞_ was validated, and clarity on the inputs into the PBPK models (e.g., adults received a single vs. multiple dose administration) (BFR02-04, BFR06, BFR14, BFR19, BFR22, and BFR26).

As with the simulated exposure depictions, providers inquired about interpreting the UAR metric. Providers asked for confirmation on their interpretation of the relationship between 95th percentile infant exposures and median adult exposures (BFR08, BFR10-12, BFR18, and BFR28). Additionally, reaffirming to themselves or with the interviewer about the magnitude of the UAR value, for instance, whether a higher UAR implies a larger risk (BFR01, BFR05, BFR11, BFR17, BFR23, and BFR24). A pediatric health systems pharmacist appeared to have a firm grasp on the UAR, explaining their understanding that a UAR of 0.44 represented the 95th percentile of pediatric AUC_0−∞_ being 44% of the median adult AUC_0−∞_, and compared the value to an RID of 15% to realize that the UAR has a larger emphasis on outlier infants (BFR03).

##### 3.2.4.2. Several factors not apparent: specify inclusion of factors in further cases and scenarios

Several factors that can be accounted for in the UAR were frequently requested by the healthcare providers, likely because the provided case scenario did not demonstrate the UAR’s ability to include various circumstances. Discussed factors included Co-medications Not Apparent [C81], *In utero* Exposures Not Apparent [C83], Metabolites Not Apparent [C86], Multiple Administrations to the Mother Not Apparent [C87], and Prematurity Not Apparent [C93] (Table 3).

First, there was a request to account for a combination of medications a mother might be taking, for example, three co-medications affecting essential nervous systems (BFR01). Second, in recognizing that infants after birth may have significant exposure to both the medication through breast milk and passed *in utero*, it was essential the latter to be accounted for (BFR03). Third, when important, drug metabolites were suggested to be incorporated into the UAR (BFR03). Fourth, one provider noted the high likelihood that mothers would be taking medications regularly and thus multiple dose regimens should be addressed (BFR22). Fifth, the need to consider prematurity and increased vulnerability at different gestational ages was expressed (BFR01).

To overcome these apparent disadvantages, a fuller explanation of the different possible scenarios the UAR can cover would be necessary. Moreover, a range of case scenarios with each of the mentioned factors [Separate by Specific Cases and Scenarios; C108] could be provided to show the UAR’s capabilities (Table 3). In creating distinct scenarios for each factor, providers suggested various scenarios including infants of different gestational ages and with specific vulnerable conditions (renal and liver disease); mothers with single vs. multiple administrations; the presence and absence of transplacental passages; and metabolite exposures. Having the UAR metric calculated for additional variables, such as breastfeeding infant ages beyond 12 months, different maternal drug doses, and a relative comparison of several different drugs (e.g., psychiatric drugs or anticonvulsants) would further the understanding of potential variables the UAR could incorporate (BFR07 and BFR10).

##### 3.2.4.3. Potential to appear subjective or misinterpreted: improve explanations on metric development and its advantages

Some healthcare providers had concerns that the UAR has Potential to Appear Subjective or Misinterpreted [C92] (Table 3). In terms of subjectivity, one provider was concerned that the predicted exposures across infant age groups may encourage delaying breastfeeding until exposures reach a level deemed safe which was thought to be impractical (BFR05). Another concern came from a teratogen/lactation information specialist who noted the issue of not realizing the UAR already accounts for multiple elements (e.g., infant age and drug bioavailability), and thus factoring them in again can make the medication artificially appear riskier to use (BFR07). A neonatologist who grasped the benefits of breast milk had apprehensions that the results of the UAR would immediately prompt a provider to advise withholding breastfeeding without further considerations (BFR14).

As measures to reduce potential subjectivity and misinterpretation that lead to negative outcomes, providers suggested to Explain More About How the Model was Made (Inputs and Assessments) [C97] and Explain More About UAR Advantages [C98] (Table 3). One suggestion was to present a deeper explanation about how each factor was weighted into the UAR, for instance, the importance of infant age playing a role in influencing the UAR (BFR05). Additionally, providing information (i.e., in the form of a table) that showed variables the UAR includes and excludes would portray which factors have been already accounted for and how they make the UAR advantageous (BFR01, BFR07, BFR10, and BFR27). Breaking down the UAR value into an understandable format by showing how each piece was determined was also thought to be helpful (BFR25).

##### 3.2.4.4. Difficult to understand or too complex: provide guidance and rationale for using the metric

At times, healthcare providers found the UAR Difficult to Understand or Too Complex [C82] (Table 3). This difficulty was commonly exhibited in the pathway to understanding the UAR. Although expressed across professions and specialties, physicians appeared more likely to express this disadvantage. Providers tended to note the complexity and complicatedness of the UAR and its potential to overwhelm and confuse others with too much information (BFR02, BFR05, BFR07, BFR18, BFR26, and BFR27).

Providing guidance and clear rationales for using the UAR would be an effective method to overcome the lack of understanding and overwhelming complexity of the novel metric. Providers postulated several strategies which were to Provide Guidance to Interpret the UAR [C106], Make Visual Representation Essential [C102], make the metric and path to its use audience-dependent, and Provide a Definitive Bottom Line [C104] (Table 3).

A guide to interpret the current presentation of the UAR was often requested by providers. The guidance would be on what each UAR value may imply, for example, if it were 0.44. Providers gave a variation of ideas to approach guidance including informing values when they would be problematic, displaying a colour-coded scheme from dangerous to minimal concern, constituting values to interpret as high vs. low exposure, and giving cut-off values with recommendations of action (e.g., through a well-devised algorithm system). Several providers valued the visual aspect of the UAR and reinforced the colour-coding concept to define potential risk.

A dichotomy became apparent in the way the UAR was preferred to be presented to healthcare providers [User Friendly for Non-Pharmacists; C100 and User Friendly for Pharmacists; C101]. Non-pharmacist providers were more inclined to only have a basic understanding of the UAR and have it presented in a simpler format that would require minimal time to provide a binary, yes or no, recommendation for the maternal-infant dyad (BFR01, BFR02, and BFR23). In contrast, it was suggested that pharmacist providers receive more detail about the UAR for a deeper understanding (BFR01 and BFR22). There were also notable nuances to the two distinct suggested approaches. Some providers suggested that regardless of the profession, having a shorter and longer form version of the UAR could be tailored to those who want a quick answer and those who tend to be more inquisitive, respectively (BFR02 and BFR09). Variation also existed within the pharmacy practice. One health systems pharmacist trained in pediatrics thought the current presentation of the UAR was appropriate (BFR22). However, another pharmacist specializing in the emergency department preferred the learning component to be thorough and once trained and familiar, an easily accessible quick version would be welcome (BFR09). One community pharmacist felt that the distinction between a less and more complex version of the UAR depended on the busyness of their practice (BFR16).

Having a definitive bottom line was a suggestion divided among providers. On one hand, providers wanted a format akin to the outdated FDA Pregnancy Categories or Hale’s L1-5, a numbering system from 1 to 10 with 10 being high risk to the infant, or an ultimate thumbs up or down (BFR06, BFR11, BFR15, BFR17, BFR18, BFR25, and BFR28). On the other hand, providers recognized the downside to providing a definitive bottom line. One pediatrician explained that categorization would make advising easier, however, there was utility in moving towards an approach to presenting the information and having the provider make the decision (BFR20). Other issues to definitive bottom lines more generally were examined in section 3.2.2.8.

##### 3.2.4.5. Lack of maternal perspective: provide a greater maternal emphasis

Although only one provider perceived the UAR to have a Lack of Maternal Perspective [C84] (Table 3), this viewpoint warranted a closer examination. For the provider, the metric seemed to focus only from the infant perspective without weighing the maternal perspective (BFR05). Therefore, it was suggested to Provide a Greater Maternal Emphasis [C105] to ensure that maternal health was also an important factor in the advising process (BFR05).

##### 3.2.4.6. Limited information on adverse effects (exposure-response relationship): provide prospective predictive evidence

A commonly cited disadvantage of the UAR by healthcare providers was Limited Information on Adverse Effects (Exposure-Response Relationship) [C85] (Table 3). Essentially, information on potential effects on the infant were described as limited with the UAR. Observations by providers included not knowing if the exposure of the medication to the infant would be harmful, having a lack of toxicity information, and the need for a clinical correlate with the UAR values. To supplement the UAR and its prediction of the dose-exposure relationship, providers suggested to Provide Prospective Predictive Evidence [C107] (Table 3). For instance, conducting prospective studies to see if the UAR would be predictive of any effects in infants (BFR03 and BFR14). Another provider explained that buy-in in their department would consist of showing that basing decisions off the novel metric would alter patient outcomes (BFR09).

##### 3.2.4.7. Not enough for clinical decision-making: combine the metric with another resource

Another commonly coded UAR disadvantage was Not Enough for Clinical Decision Making [C88] (Table 3). For many providers, the UAR alone would not convince them to immediately change practice. Instead, the need to compare results of the UAR with other resources was necessary (BFR01). Providers also had concerns that the difficulty of explaining the UAR to the family would be an obstacle to incorporate the metric into practice (BFR18). One family medicine physician clearly voiced they would not use the metric alone to make a medical decision and valued existing resources that compiled evidence from all existing studies to provide guidance (BFR19).

The proposal to Combine the UAR with Another Resource [C96] was mentioned by multiple providers (Table 3). In their views, adding the UAR to an existing informational resource such as LactMed or MMM would be beneficial and having both the novel metric and summary of the existing scope of evidence would give confidence to use the UAR (BFR01, BFR02, BFR04, BFR05, BFR07, BFR10, BFR11, and BFR27). Incorporating the UAR to an existing informational resource could also assist with access to the novel metric (BFR08). Alternatively, one provider suggested incorporating the most useful sections of existing informational resources into the UAR (BFR17). Nevertheless, results from this code suggest that the UAR is presented as a complementary piece within commonly used resources as illustrated in Figure 1.

##### 3.2.4.8. Unusable in its current form (too novel): simplify, train, and educate to reduce effects of novelty

Healthcare providers frequently voiced that the UAR was Unusable in its Current Form (Too Novel) [C94] (Table 3). Generally, providers felt that the period of time they were exposed to learn about and use the novel metric was too short (BFR06, BFR07, BFR10, BFR13, BFR14). More assistance would be needed to interpret the UAR to feel comfortable with its use (BFR18). The UAR was also too novel for immediate uptake and providers needed more experience with it (BFR10, BFR20, BFR21, BFR25, and BFR27).

Provider suggested strategies to Add a Summary Statement [C95], Give Specific Training [C99], and Overcome Simulation Skepticism [C103] for alleviating concerns about the current form and novelty of the UAR (Table 3). A summary statement for the UAR was imagined as a common sense recommendation to translate the UAR results so that they are practical and understandable (BFR02, BFR15, BFR19, and BFR23).

Giving specific training about the UAR was a widely discussed strategy to improve the UAR. Providers had different suggested methods for training including providing course lectures and presentations, targeting training to departments for improved uptake, incorporating the metric into educational programming (i.e., pharmacy education), and conference talks and seminars. Related to training was a suggestion to overcome provider simulation skepticism. One provider described that the simulation component could be difficult to trust and understand and thus giving more education on this topic would help with UAR uptake (BFR01). Another provider suggested educating others on the idea that PBPK modeling is not a novel approach and is in fact a method commonly used in drug development and FDA approvals (BFR03).

## 4. Discussion

Our paper sought to address the question, among healthcare providers advising mothers taking medications while breastfeeding, whether the UAR will confer benefits over existing resources and whether improvements for optimal uptake could be attained. We were interested in how resources are currently being used, whether there is a need for the UAR in addition to current resources, how the UAR could be used in practice, whether the UAR would confer benefits, which healthcare providers would particularly benefit from use of the UAR, and how the UAR could be further improved for clinical practice. To investigate these questions, we used one-on-one semi-structured interviews with healthcare providers followed by the Framework Method strategy of analysis. Results of our work are highlighted in the following main findings. First, informational and metric resources are used as one of three tactics in current advising practices, with two other methods being clinical experience and identifying a need for referral or consultation. Second, based on the number of disadvantages of existing resources that can be addressed and supplemented by the UAR, we have deemed there to be a need for improvement of current resources and that the UAR would confer benefits. Third, the UAR in its current state would most benefit from use as a complementary piece within commonly used resources, such as LactMed and MMM. Fourth, although providers valued the format of the UAR to be dependent on profession, results suggest that providers across professions and disciplines would benefit from UAR use. Fifth, through the interviews, we were able to identify multiple strategies to improve the UAR for clinical practice.

Through examining current practice approaches, a workflow that healthcare providers typically followed in their practice was identified (Figure 1). This workflow served as a backbone that related all other aspects and themes of advising. Three main approaches were discovered as informational resource use, clinical experience, and identify need for referral or consultation. The most exercised approach by providers was Resource Use as a First Go-to [C58]. The use of informational resources appeared valuable, including its application to evaluate the quality of evidence and conducting risk-benefit analyses. Our study expands on a pilot study by Byerley (20), which indicated that pharmacists reported use of a wide range of resources such as UpToDate, LactMed, and MMM. We confirm this finding and categorize the resources as general and lactation-specific.

Three findings regarding current practice approaches were found to be unexpected. First, the act of referrals occurred rather frequently with community pharmacists. These providers felt that when presented with a case for which they did not feel able to adequately provide a recommendation, other providers would be consulted. Community pharmacists are encouraged to play a greater role in maternal health services, including providing breastfeeding guidance, however, their extent of practice in this area needs to be strengthened (15, 21). Our results indicated that community pharmacists would be better equipped to advise breastfeeding patients if some of the hurdles of advising were overcome, including access to lactation-specific resources. Second, our study was the first to examine resource metric use and found that there was a universal lack of overall application among interviewed healthcare providers. When asked about common metrics such as the RID and Hale’s L1-5 categories, most providers were unfamiliar with them or not sufficiently confident to use them in their practice. Third, there was a prevalent use of FDA Pregnancy Categories by interviewed healthcare providers. As Burkey and Holmes (22) explain, these categories are often confusing and misleading, and moreover, not intended for use in lactation.

Exploring the disadvantages of existing resources uncovered several shortcomings. Accessibility was identified as the most cited disadvantage to some resources and affected healthcare provider perception and use of the resource. Through the interview process, it became clear that many advantages of existing resources and the UAR have potential to overcome disadvantages of current resources. Although not an intended outcome from this study, asking questions regarding existing resources and subsequently the UAR assisted in the comparison of existing resources with the UAR. Providers were able to critically identify specific disadvantages that the UAR may address and vice versa. For example, reflecting on an advantage of the UAR reminded the provider that current resources are unable to address this advising need and thus deemed it as a disadvantage to existing resources.

Several disadvantages of the UAR were revealed and healthcare providers identified strategies to overcome its limitations. An important finding from the interviews was that the UAR in its current state would not be used alone for clinical practice. The complexity of the UAR was a main barrier to use. Two additional notable influences include Potential to Appear Subjective or Misinterpreted [C92] and Limited Information on Adverse Effects (Exposure-Response Relationship) [C85]. These codes were likely acknowledged since the interviewed providers were generally well-versed in resource use and had many years of advising experience. Additionally, due to almost all providers belonging to a Pro-breastfeeding Culture of California [C30], and San Diego in particular, it would be fitting that there are concerns about the UAR in increasing the likelihood of inappropriately advising against breastfeeding. Nonetheless, these shortcomings signal the importance of considering the culture and environment of practice, and the importance of the educational and training aspects of the UAR. Thus, developed training of the UAR should account for the environment of practice and speak to issues regarding the exposure-response relationship. An example of addressing the latter would be explaining that although the UAR does not directly assess drug response, it does account for the idea that some breastfed infants may get to adult therapeutic, and potentially supratherapeutic, exposures. With this understanding combined with knowledge of the mechanism of action and toxicity in adults, clinicians would be better poised to make more informed assessments. Improved training on resource use generally should improve practice, especially since previous work has found that provider knowledge and training can influence their interpretation of drug risk (23).

This study is a prime example of gathering information from potential end-users on a novel tool in order to identify targeted areas of improvement to ensure future optimal use. Our work was the first to compile rich information on advantages and disadvantages of currently used resources from end-users. The gathered information was insightful and could be directly applied to identify gaps for UAR improvement. As another strength of the study, we recruited and interviewed a broad range of professions and specializations. Therefore, our interview findings were from a diverse range of role and discipline perspectives that could be compared. However, other than profession and specialization, our participants tended to be uniform in other demographic areas. Consequently, we could not discern meaningful patterns across other variables, such as gender identity, race/ethnicity, and level of advising experience. Additionally, it should be noted that the use of snowball sampling led to providers recruited from similar institutions with comparable practices and perspectives (e.g., pro-breastfeeding and advanced users of lactation-specific resources). Accordingly, we were unable to receive a direct understanding from providers who practiced a Culture of Leaning Towards Caution [C29] to gain more insight on their current practice approaches and viewpoints on existing resources and the UAR.

To further our understanding and improve the uptake of the UAR, future studies are suggested. First, a study to understand how to optimally provide training to providers in each profession and discipline for both existing resources and the UAR would be valuable. Second, research into the use of various existing resources such as the package insert and general drug information databases (e.g., Micromedex, Lexicomp) would help clarify their potential on decision making in order to improve provider knowledge and confidence in advising breastfeeding mothers during medication use. Finally, it would be of interest to improve the UAR based on this study’s findings (e.g., Combine the UAR with Another Resource [C96] and Provide Guidance to Interpret the UAR [C106]) and perform another study to assess whether the needed improvements were adequately addressed to ensure optimal use.

## Data Availability

The datasets presented in this article are not readily available because of participant confidentiality outlined in the ethics approval process. Requests to access the datasets should be directed to aedginto@uwaterloo.ca.
