# Supplementary material for: Addressing maternal medication use during breastfeeding using clinical resources and a novel physiologically based pharmacokinetic model-derived metric: A qualitative study
Source: Front Pediatr. 2023 Apr 3;11:1147566. doi: 10.3389/fped.2023.1147566 (PMC10106747; doi:10.3389/fped.2023.1147566)
Supplement: Supplementary file 1 [file Table1.docx]

Supplementary Material

Addressing maternal medication use during breastfeeding using clinical resources and a novel physiologically-based pharmacokinetic model-derived metric: A qualitative study

Cindy Hoi Ting Yeung^1^, Sherilyn K.D. Houle^1^, Philip O. Anderson^2^, Brookie M. Best^2^, Samuel Dubinsky^1^, Andrea N. Edginton^1*^

^1^School of Pharmacy, Faculty of Science, University of Waterloo, Kitchener, Ontario, Canada

^2^Skaggs School of Pharmacy and Pharmaceutical Sciences, University of California San Diego, La Jolla, California, U.S.

*** Correspondence:**Andrea N. Edginton, [aedginto@uwaterloo.ca](mailto:aedginto@uwaterloo.ca)
10A Victoria St S, Kitchener, ON N2G 1C5
Tel: (519) 888-4567 ext. 84408

# Interview Guide

#
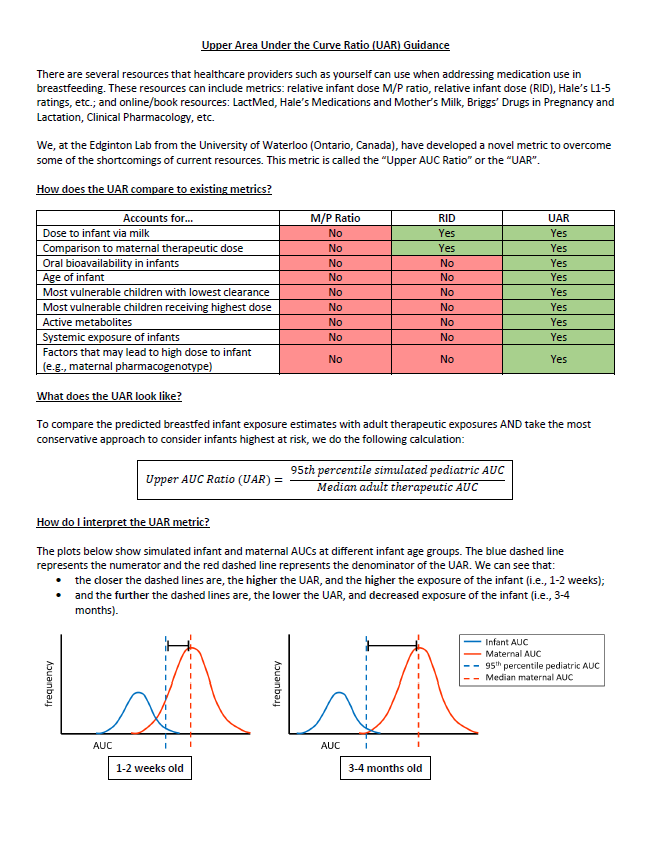


#
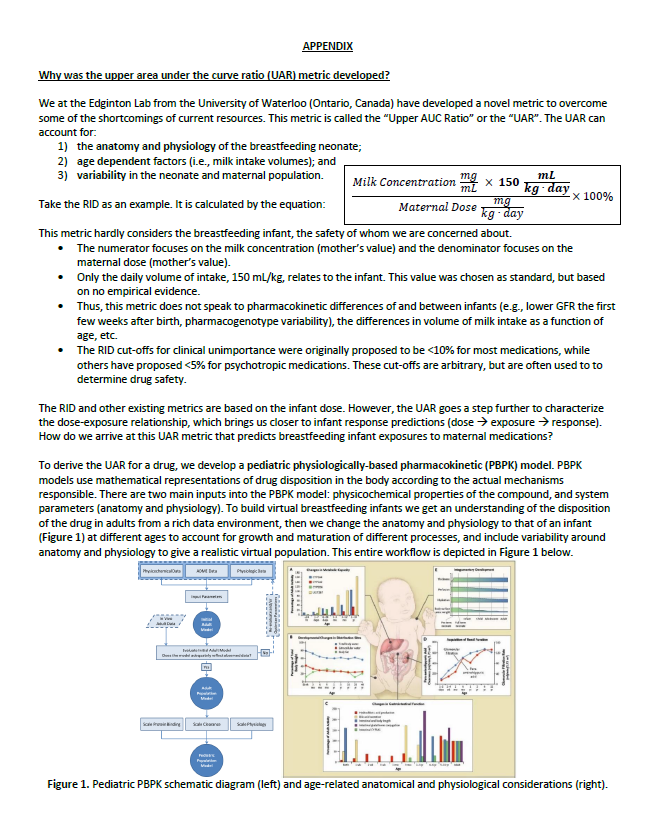


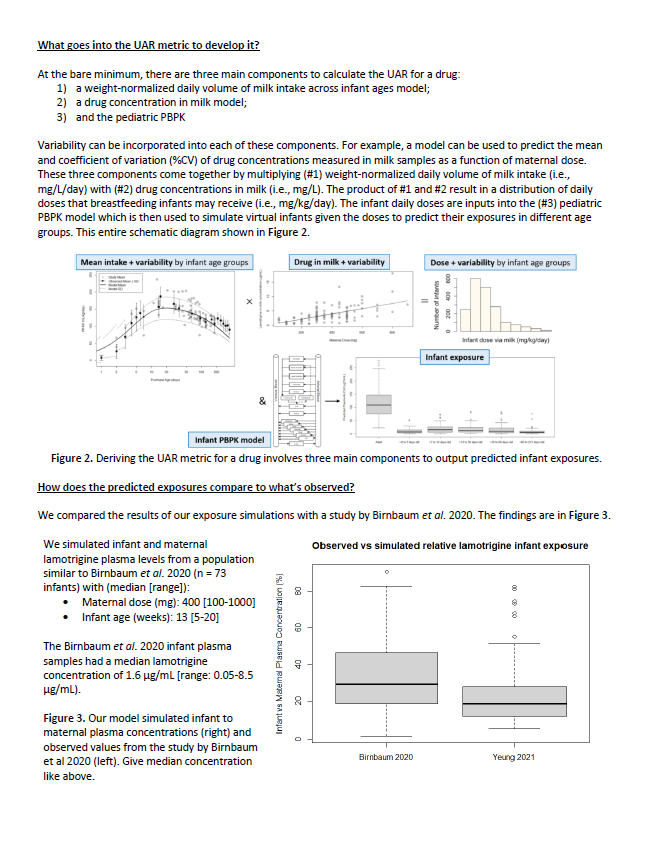


**Note:** The caption for **Figure 3** was presented to participants as shown above. However, for more clarity, it should be read as: “**Figure 3.** On the right are the simulated infant to maternal plasma concentration ratios from our model, and on the left are the observed ratios from the study by Birnbaum *et al.* 2020.”


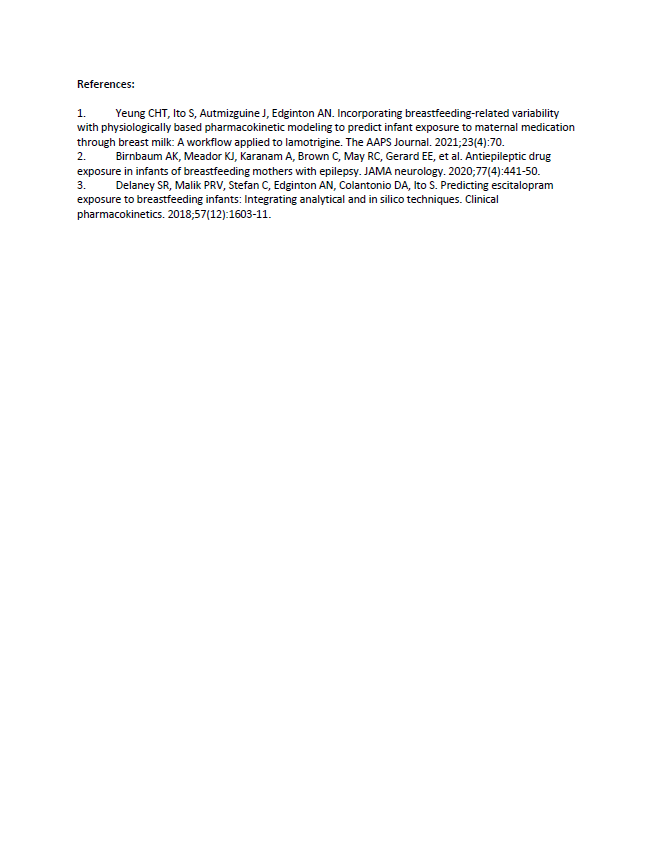


##
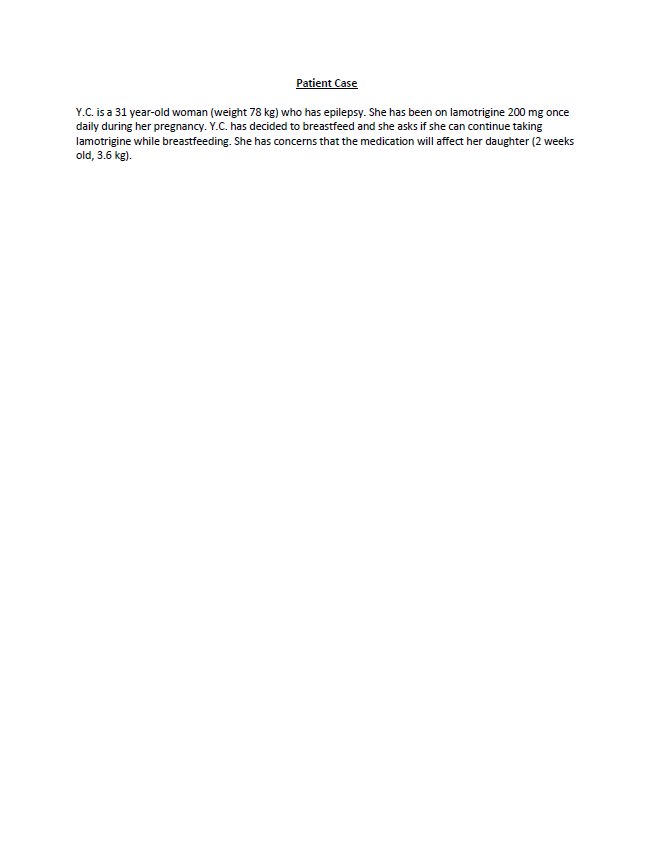


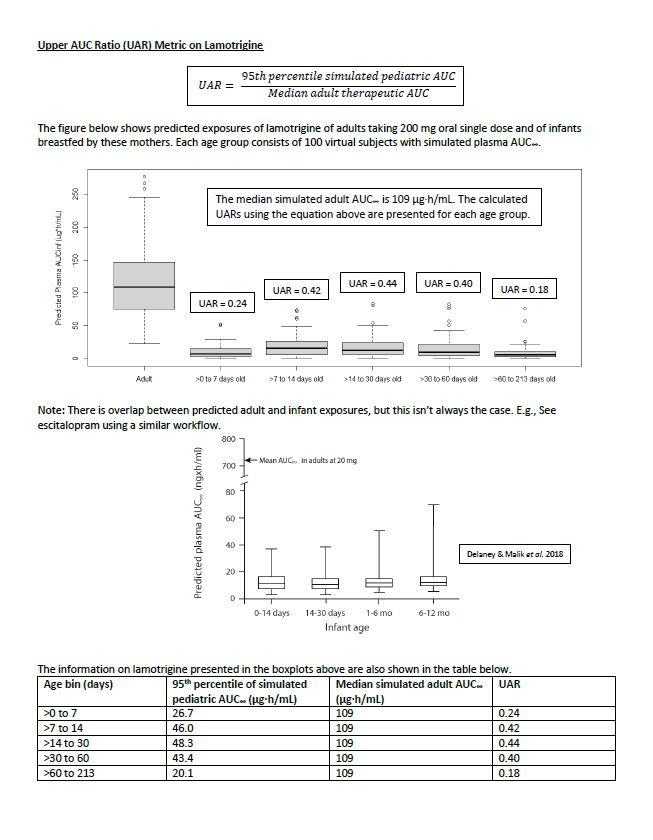


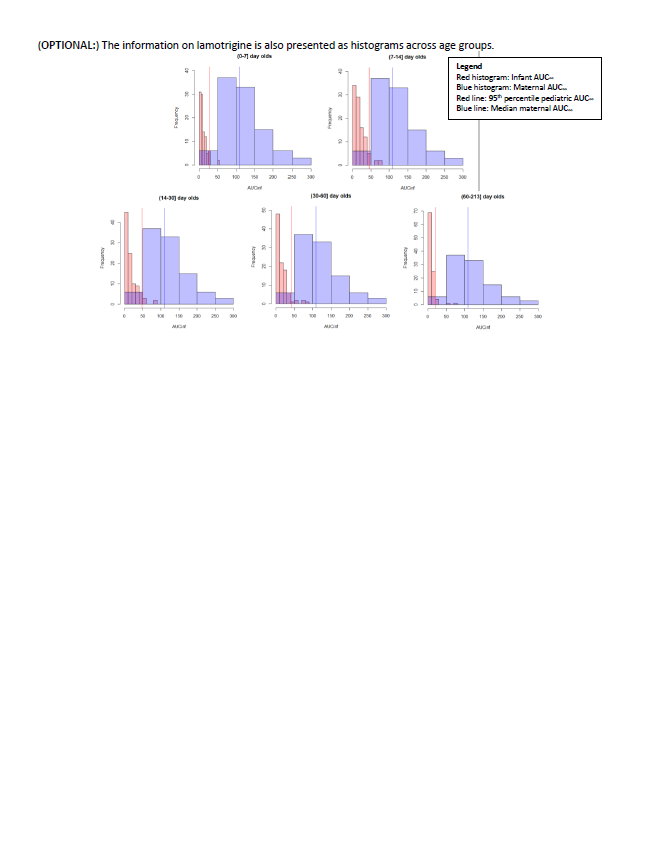


# Interview Materials: New Metric Guidance and Presented Scenario

The one-on-one semi-structured interviews will cover the following broad components:

1. Discuss current practices regarding which resources are considered when addressing drug use in breastfeeding.

Question: When addressing drug use in breastfeeding, which resources (metrics and online/book resources) do you currently access?

Question: Can you walk me through how you use these resources when addressing drug use in breastfeeding?

1. Provide scenarios and existing resources, asking participants how they would proceed in practice.

Question: Given the following scenario and the resources (metrics and online/book resources) that you are currently using, can you outline how you would proceed in practice?

1. Present the same scenarios, but provide the UAR and ask if it impacts how the participants would proceed in practice.

Present material to the participant that explains the novel metric, the upper area under the curve ratio (UAR).

Question: With new information about the UAR and given the same scenario as before, how would you now proceed in practice?

1. Discuss the advantages and disadvantages of each method used in steps 2 and 3.

Question: Can you describe some advantages of each current resource (metrics and online/book resources) that you used in the scenario? What about some disadvantages?

Question: Can you describe some advantages of the UAR for use in your current practice? What about some disadvantages?

1. Seek suggestions on how the UAR can be improved or described to clinicians to ensure it is most useful to them as end-users.

Question: Given the disadvantages of the UAR that you mentioned, what are some actionable suggestions to improve or better describe the metric to other clinicians?

# NVivo Code Book Output

| Name | Description |
| --- | --- |
| T1: Advantages of Existing Resources |  |
| C1: Accessible Through the Institution | Resource is readily available and cost is not a barrier due to the institution the provider is working at. |
| C2: Comprehensive | Resource is thought to contain a wide range of information which may include reporting of risk metrics, drug physicochemical properties and ADME, and multiple referenced studies. Healthcare provider may consider the resource to be sufficient. |
| C3: Distinguishes and Provides Various Types of Data | Resource includes and differentiates between data types which can include animal versus human, case studies versus larger cohort, maternal milk levels versus infant plasma and/or adverse reactions, etc. These data types may reflect distinguishing "strong versus weak" evidence. |
| C4: Evidence to Support Use | Studies have been conducted on the resource to support its use in practice. |
| C5: Familiarity | Resource is recognizable by the provider, colleagues, and/or the field. |
| C6: Generally Accessible | Resource is readily available, easy to use, and factors such as cost, cell phone compatibility, and physical copy versions are generally not a barrier to use. |
| C7: Patient-friendly | Resource can be easily explained or directly shown to patients. |
| C8: Summarizes and References Evidence | Resource incorporates and may reference available evidence in their contents. |
| C9: Summary Statements | Resource provides a summary statement which may summarize available evidence in a few sentences. |
| C10: Trusted Authors | Authors and/or administration/board overseeing the resource is trustworthy. |
| C11: Up to Date | Resource is updated and disseminated regularly. |
| T2: Advantages of the UAR |  |
| C12: Addresses Clearance Differences | Metric is able to differentiate between individuals with differences in clearance, for instance, preterm vs term infants, hepatically impaired infants, etc. |
| C13: Addresses Exposures (AUC) | Incorporates a consideration of infant (and/or maternal) exposures to the drug (AUC). |
| C14: Addresses Multiple Considerations | Metric considers several factors, such as listing the following as advantages: infant age, maternal variables (e.g., pharmacogenotype), and infant exposure. As a result, the metric can be thought of more individualized and specific to situations. |
| C15: Addresses Scarcity of Published Information | Metric has potential to use the existing paucity of data to help providers make more data-informed decisions (i.e., through PBPK modeling, only requiring confirmatory rather than exploratory samples), whereas, current resources are unable to extrapolate and rely on direct conclusions of few published literature. |
| C16: Addresses the Age of the Infant | Incorporates a consideration of infant age into the metric. Can include considerations on volume of intake, colostrum levels, etc. as a function of infant age. |
| C17: Addresses the Maternal-infant Pair | Incorporates a consideration of both the mother and infant into the metric. |
| C18: Addresses the Worst Case Scenario | Incorporates a consideration of the worst case scenario (i.e., outliers highest at risk) for breastfeeding infants. |
| C19: Can Share with Other Providers and Patients | Metric can be shared with other healthcare providers and/or patients for their understanding. |
| C20: Numerical Metric | Metric is presented as a number (i.e., a ratio) that is useful in advising. |
| C21: Objective | Metric is not subjectively derived. |
| C22: Opens Up the Thought Process | Healthcare provider uses the metric to consider factors they may have not considered with current resources. As examples: going beyond dose considerations, thinking through a process involving exposures and risk to the infant, and reflecting on the contents of the Table that compares the UAR with RID and M/P ratio. |
| C23: Understand Existing Observations, Evidence, and Recommendations | Metric can help elucidate observations (i.e., breastfed infants typically do not have adverse reactions with their patients taking lamotrigine), evidence (i.e., literature describes volume of intake and thus risk to be higher at 2 weeks), and recommendations (i.e., typically leaning towards advisign to breastfeed) used by the healthcare providers in their current practice. |
| C24: Visual Representation | Metric is presented visually in a pictorial, flowchart, and/or graph (histogram or boxplot) that is helpful. |
| T3: Current Practice Approaches |  |
| C25: Advise a Cautious Approach | A personal approach to be more cautious when advising to breastfeed (i.e., recommending to stop/refrain/"pump and dump") by the interviewed healthcare provider. |
| C26: Advise to Breastfeed During Medication Use | A personal approach that tends to recommend breastfeeding by the interviewed healthcare provider. |
| C27: Approach for Lack of Evidence | Healthcare provider has additional resources they may use when the drug has sparse information. |
| C28: Continue Medication as a First Go-to | Main first step of provider is to see if patient was taking medication during pregnancy. |
| Culture of Practice |  |
| C29: Culture of Leaning Towards Caution | A remark regarding a tendency to recommend stopping/refraining/"pumping and dumping" at the institutional/state level which can involve a broad reference to other practitioners. |
| C30: Pro-breastfeeding Culture of California | A remark regarding a tendency to recommend breastfeeding at the institutional/state level which can involve a broad reference to other practitioners. |
| C31: Evaluate the Quality of Evidence | Healthcare provider typically reviews and considers the existing published evidence which can be as provided by the resource. |
| Factors to Consider |  |
| C32: Alternative Pharmacological Class | In the healthcare provider's advising process, considering another drug with a similar effect (i.e., with less known risks to the infant). |
| C33: Drug Physicochemical and ADME Properties | Healthcare provider uses the physicochemical properties (i.e., molecular weight, lipophilicity) and ADME (i.e., Tmax, bioavailability) characteristics of the drug in their advising. |
| C34: Drug Use in Pregnancy | Healthcare provider uses the fact that the patient was taking the drug during pregnancy in their advising (beyond a check as the first step in their advising). |
| C35: Health of the Infant | Needing more information on, or giving consideration to, the health state of the infant (preterm, co-morbidities, monitoring for adverse reactions, etc.). |
| C36: Health of the Mother | Needing more information on, or giving consideration to, the health state of the mother (has an underlying condition, etc.). For instance, asking whether the mother needs the medication if they are not doing well health-wise. |
| C37: Information on the Drug Used in Infants | Whether the drug has reports of being directly administered in infants. |
| C38: Maternal Co-medications | Whether the mother is current taking additional medications. |
| C39: Maternal Dose Taken | Dose of the drug of interest that the mother is taking. |
| C40: Risks and Benefits (Analysis) | Healthcare provider goes into depth (making a thoughtful assessment) about weighing the risks and benefits. For example, giving an antibiotic to the mother if the risk to the infant is getting diarrhea versus benefits of breastfeeding. |
| C41: Select Drug Cases for Non-Resource and Resource Use | Healthcare provider is selective on using resources because their cases can be on specific reoccurring drugs where they use experience and previous knowledge. They discuss distinct situations about making decisions without needing resources and situations where resources are needed. |
| C42: Team Approach (Present or Absent) | The presence or absence of a team approach to advising is taken, which may reference situations where multiple healthcare providers are or are not involved. |
| C43: Time of Breastfeed Relative to Dose | Healthcare provider considers timing of when breastfeeding occurs along in relation to dose administration in their advising. |
| C44: Type of Breastfeeding (Exclusive vs Partial) | Knowledge of the mother's state of breastfeeding as exclusive or partial. |
| C45: Lack of Existing Metric Use | Healthcare provider notes lack of metric use (Hale's L1-5, RID, M/P ratio, etc.) in their advising. |
| C46: Multiple Resource Use | Mentioning multiple resources that are accessed (i.e., in addition to naming a primary resource). Includes metrics (e.g., RID) as well, for instance, mentioning use of the RID and another resource such as LactMed. |
| C47: Pregnancy Categories (Using or Avoiding Them) | Mention of pregnancy categories applied in practice. Either the interviewed healthcare provider uses them or avoids them as a personal approach. |
| C48: Primary versus Secondary Resources | Healthcare provider appears to use a distinct primary resource and more secondary resources. Identifying the primary and secondary resources may suggest multiple resource use. |
| Realities of Advising |  |
| C49: Concern for Liability | Healthcare provider liability mentioned as a factor to consider when advising patients. |
| C50: Concerns Relaying Evidence-based Decisions | Concern that an evidence-based decision (i.e., patient recommended to breastfeed) is not relayed to other healthcare providers in the patient's care. |
| C51: Institution Needs Resource Justification | Healthcare provider can only attain a resource if provided enough justification to their employed institution. |
| C52: Lack of Information About the Patients | Healthcare provider has limited access to information about the mother and infant (i.e., their patient records). |
| C53: Minimal Time for Clinical Decision Making | Limited time to advise a patient is remarked as a reality in practice and potentially a barrier to resource use and thorough decision-making. |
| C54: Motives of Manufacturers | Drug manufacturer motives are a factor in advising, such as, the source of minimal drug in lactation studies and studied adverse effects in infants. |
| C55: Variable Patient Health Literacy | Patient health literacy is noted as a barrier or simply a factor to advising (e.g., in explaining the recommendations of a resource that is above the Grade 6 reading level). |
| C56: Refer to Other Provider | Preference of the interviewed healthcare provider to refer to another provider (e.g., physician) typically for decision-making and risk assessment. |
| C57: Reliance on Other Provider or Resource | Healthcare provider tends to rely on assessment or advice of another provider (e.g., lactation consultant) or via resource (e.g., LactMed due to trusted authors, Hale's due to author already calculated or assessed metrics). Excludes referrals to another healthcare provider (i.e., physician) where limited decision-making and assessment is made by the interviewed provider. |
| C58: Resource as a First Go-to | Main first step of provider is to access a resource. |
| C59: Use Combination of Experiences and Resources | Healthcare provider uses their experience with advising (e.g., familiarity with drug) and additional resources such as the RID or Hale's. |
| C60: Use of Package Inserts | Mention of product monograph package insert information on the drug in lactation use in advising. |
| Use of RID in Specific Cases |  |
| C61: Comparing within Drug Class | RID is specifically used when comparing drugs within a class. For example, recommending a drug in the same class with a lower RID value. |
| C62: Explain a Range of Outcomes in Infants | RID is used to explain why infants experience a range of outcomes, from no side effects to visible adverse events. Mentions how much of the dose an infant might receive. |
| C63: Mother on Co-Medications | RID is considered when the mother is on co-medications. For instance, a mother taking multiple medications and health is at risk, and drug of interest has high RID may lead to a risk vs benefit assessment. |
| C64: Mother with Conditions | RID is considered when the mother has comorbidities. For instance, a mother taking has multiple conditions and health is at risk, and drug of interest has high RID may lead to a risk vs benefit assessment. |
| C65: New Medication | RID is used when a new medication is presented with sparse information or healthcare provider has minimal familiarity. |
| C66: Reassurance Along with Other Resources | RID is used in combination with other resources (e.g., LactMed) to reassure the healthcare provider's assessment and recommendation. |
| T4: Disadvantages of Existing Resources |  |
| C67: Areas of Subjectivity | Resource is noted to lack objectivity in their contents and conclusions. |
| C68: Co-medications Not Considered | Resource does not consider cases where mother and/or infant are exposed to multiple medications. |
| C69: Easily Outdated | Resource can easily be out-dated and does not necessarily present the most up-to-date information. |
| C70: Effect on Milk Not Considered | Resource does not consider that the medication has an effect on milk (composition, production, etc.). |
| C71: Inaccessible | Resource is not easily accessed generally or through the healthcare provider's institution. Barriers to use can include cost and difficulty attaining the resource (i.e., physical book copy or needing to go through several webpages). |
| C72: Infant Age Not Considered | Resource does not consider the age of the infant. |
| C73: Maternal Dose Not Considered | Resource does not consider the dose administered to breastfeeding mother. |
| C74: Non-average Cases Not Considered | Resource does not consider outliers, or maternal-infant pairs who are not the average case. |
| C75: Overreliance on a Single Resource | Concerns that when healthcare providers over rely on certain resources (Physicians Desk Reference, existing metrics, etc.), they can lead to negative consequences (i.e., recommending not to breastfeed when the drug is actually of low risk according to other resources). |
| C76: Overreliance on Case Reports and Published Data | Relying on scantly presented information of a single case report or minimal published data (whether due to universal lack of information on the drug, or the resource does not include all published data). |
| C77: Perceived Lack of Reported Information Due to a Resource | As a result of the resource not including the full available information for advising. Not necessarily a direct result of no data or information collected on the drug (i.e., lack of studies conducted), nor are specific areas specified to be lacking (e.g., lack of co-medication considerations, effect on milk considerations) as the reference is more generally missing information (i.e., the reference is generally lacking information). |
| C78: Too Broad | Presented information in the resource is not specific and does not provide useful information for conclusions. This can include the perception of healthcare provider that a concrete/specific bottom line is not provided. |
| C79: Too Much Information or Text-heavy | Resource provides too much information that may overwhelm the healthcare provider in their advising. |
| C80: Unclear Conclusions | Reference lacks a statement that summarizes the information or existing evidence. |
| T5: Disadvantages of the UAR |  |
| C81: Co-medications Not Apparent | In the provided example scenario, co-medications of the maternal-infant pair were not considered, thus appearing as a disadvantage of the metric. |
| C82: Difficult to Understand or Too Complex | Metric appears to be difficult to understand or too complex for either the interviewed healthcare provider, or in their perspective, a wider audience. |
| C83: In utero Exposures Not Apparent | In the provided example scenario, in utero exposure to the infant was not considered, thus appearing as a disadvantage of the metric. |
| C84: Lack of Maternal Perspective | Healthcare provider felt that the maternal perspective was under considered in the presentation of the metric. |
| C85: Limited Information on Adverse Effects (Exposure-Response Relationship) | Information on the effect on the infant, including adverse effects from the exposure-response relationship were limited. |
| C86: Metabolites Not Apparent | In the provided example scenario, drug metabolites were not considered, thus appearing as a disadvantage of the metric. |
| C87: Multiple Administrations to the Mother Not Apparent | In the provided example scenario, multiple dosing to the mother was not considered, thus appearing as a disadvantage of the metric. |
| C88: Not Enough for Clinical Decision Making | Healthcare provider noted that using the metric alone (i.e., without any further resources) was insufficient to make a clinical decision. |
| Path to Understanding the UAR |  |
| C89: Exposure Comparisons | When understanding the UAR and applying it to the example scenario, the healthcare provider inquired about the depictions and explanations of infant versus adult, and/or across infant ages relative exposures. |
| C90: Interpreting the Exposure Estimates | Interpreting the depictions and explanation of the maternal and infant exposures. Includes what was incorporated into producing the individual maternal and infant exposure estimates (versus the UAR value). |
| C91: Interpreting the UAR | In understanding the metric for the scenario, healthcare provider inquires on the interpretation of the UAR value (e.g., the numerical 0.44) and the use of numerator (95th percentile infant) and denominator (median maternal). It also includes inquiries on what the UAR takes into account (versus how the individual maternal and infant exposures were determined). |
| C92: Potential to Appear Subjective or Misinterpreted | Metric may be interpreted as subjective mainly due to lack of conclusive guidance (i.e., only based on provider's assessment of level of infant and maternal exposure overlap). |
| C93: Prematurity Not Apparent | In the provided example scenario, infant prematurity was not considered, thus appearing as a disadvantage of the metric. |
| C94: Unusable in Current Form (Too Novel) | More training, familiarity, and time with the metric were hinderances to its current use. |
| T6: Strategies to Improve the UAR |  |
| C95: Add a Summary Statement | Provide a summary statement with how the metric is currently presented. |
| C96: Combine the UAR with Another Resource | Present the UAR along with other resources, such as, embedded in Hale's or LactMed. |
| C97: Explain More About How the Model Was Made (Inputs and Assessments) | Description of how the model was made (the inputs and assessments) could be strengthened. |
| C98: Explain More About UAR Advantages | Explaining more about the specific advantages of the UAR over current metrics. |
| C99: Give Specific Training | Provide specific training on the UAR to healthcare providers. For example, training modules on model development and how to interpret the UAR. |
| Make the Metric and Path to Its Use Audience-dependent |  |
| C100: User Friendly for Non-pharmacists | Make the metric more user-friendly and path to being able to use it catered towards non-pharmacists. This may include providing a brief summary of the UAR and its conclusions, and yet include background on concepts such as AUC. |
| C101: User Friendly for Pharmacists | Make the metric more user-friendly and path to being able to use it catered towards pharmacists. This may include providing more detail into the development and scenarios of application. |
| C102: Make Visual Representation Essential | Ensure there is visual representation of the UAR when presenting the metric for drugs. |
| C103: Overcome Simulation Skepticism | Present the UAR in ways that reduce or prevent skepticism over developing risk estimates from simulations (i.e., virtual populations). |
| C104: Provide a Definitive Bottom Line | Rather than presenting the UAR value as it is, provide a strict bottom line. For example, categories that allow healthcare providers to more easily interpret the conclusion of the UAR. |
| C105: Provide a Greater Maternal Emphasis | In the description of the UAR, provide a larger maternal emphasis (i.e., maternal health and her needed therapy). |
| C106: Provide Guidance to Interpret the UAR Metric | Provide a guide to interpret the UAR as currently presented (e.g., what does 0.44 mean?). |
| C107: Provide Prospective Predictive Evidence | As a next step in UAR development, conduct and provide evidence that the UAR can prospectively predict infant exposure risk in practice. |
| C108: Separate by Specific Cases and Scenarios | Present the UAR according to specific cases and scenarios for the drug. For example, including in utero exposures, premature infants, different maternal doses, and in comparison with other drugs (i.e., within the same drug class). |

# Illustrative theme and code quotes

**Current Practice Approaches**

Advise a Cautious Approach

“… in all honesty, I, still err on the side of caution […] because there is still exposure.” (BFR06)

“… if the risks were […] high […] in the initial phase, maybe you can pump and dump until you got to […] 30 days or 60 days […] which would be difficult for a mom to do. But I mean it could be possible if she was really concerned about the exposure.” (BFR12)

“… if there is a safe alternative, is there a way we can pump and dump until we get that out of her system, and then how long do we have to pump and dump?” (BFR15)

Advise to Breastfeed During Medication Use

“Rather than just saying, ‘… no you had a kidney transplant, you’re on X, Y, Z, we’re not gonna let you use your milk,’ we try to really do everything we could to get that milk into the baby, or even suggest sometimes like a half and half diet to minimize the contact with something. We just didn’t understand more, but we still wanted the baby to have the biology of the milk.” (BFR02)

“This is not a very surprising dose, with the 200 mg. I’m looking at that, once a day. That’s not [a] particularly high dose, which is good. I’m glad she’s decided to breastfeed. So, I would tell her that there are real advantages to herself and her baby.” (BFR07)

“I think that there's still very few medications that I'm like, ‘Okay we know for sure that this is bad, and that this is not good for baby’, or ‘These are alternatives to this medication’, and otherwise I continue to recommend breastfeeding because I feel like we know that there are benefits of breast milk and there are lots of things that we don't know about for children and on, yet, we still do them.” (BFR15)

Approach for Lack of Evidence

“But I don't know anyone that uses M/P ratio, if that's, unless it's all like got or something. I mean the problem is, the data is not there on a lot of things.” (BFR07)

“If that feels like it's not giving me enough information, then I would probably go to InfantRisk and see if there's more information. I like InfantRisk because they also answer clinicians’ questions who submit those, so sometimes, you can kinda do a search for if somebody had a question similar to mine.” (BFR25)

Continue Medication at First Go-to

“So this woman would've been encouraged to breastfeed before anybody looks up the medication, assuming OB had decided it was safe for the fetus, and she'd be encouraged to keep breastfeeding while we make our decision.” (BFR02)

“That baby's already been exposed, so what's the, what's the harm of continuing some low-level exposure? If the baby hasn't been harmed already, by this point, by two weeks of age.” (BFR03)

“If this has been a stable regimen for her throughout her pregnancy, then thus infant's already been exposed to lamotrigine which I would use maybe as a comforting measure for her to... for her to realize that this exposure's already happening and see, her baby is here and fine.” (BFR05)

Culture of Practice: Culture of Leaning Towards Caution

“And I've had this happen with medication. Then we contact them and say the, the patient has been told that she can. A lot of patients have been inappropriately told that they cannot breastfeed with a lot of these meds that are actually okay, that's the problem. And they say, ‘Oh, I didn't. I haven't breastfeed my three other kids 'cause I've been on tegretol for the last three pregnancy.’ So de-mything that, quite honestly, and convincing them that it's safe is sometimes difficult.” (BFR08)

“… if I have adult providers prescribing something for my moms, they often will tell them to, like, pump and dump or not use their breast milk. […] They just prescribed like an antibiotic for mastitis or […] bronchitis or something, […] so we have to do some education about that, and I can pull up LactMed to show the moms that it's safe...” (BFR15)

“… many of them will admit, I'm just so busy, I don't have time, and it's just easier to say don't do it when we know that most medications are compatible with breastfeeding.” (BFR27)

Culture of Practice: Pro-breastfeeding Culture of California

“I think some of the NICU providers our age tend to be like just pro breast milk period.” (BFR15)

“… some clinicians would have some caution. But, that most babies don't have side effects. That there's a known benefit to breastfeeding, and that can offer, if there's any guidance on what to look for side effects for the baby.” (BFR21)

Evaluate the Quality of Evidence

“So […] these different resources will refer to other literature, but then I also wanna know is one referring to a paper of a case report of two patients versus another one is referring to a really extensive, well-designed whatever PK study in […] 30 patient[s]. Like, so I also look at the quality of evidence they're quoting and then I gear towards the ones that are higher quality.” (BFR04)

“… if [information from resources are] different, but I have more evidence for this one than I do this one, then I'm gonna lean towards the one that I actually have evidence 'cause then I can personally take that evidence and make my own clinical decision.” (BFR25)

Factors to Consider: Alternative Pharmacological Class

“and if there's other options, 'cause that would be a discussion with the neurologist, I think, taking care of her.” (BFR22)

Factors to Consider: Drug Physicochemical and ADME Properties

“And lamotrigine is one of those agents that the amount of metabolism varies tremendously. I mean, the amount, the levels and the amount of milk and it's believed to be based on maternal pharmacogenetics.” (BFR07)

“Generally, if something is over that 500 daltons, it doesn't get absorbed very well into the milk or across the placenta. So, one of the common ones that we get is the different, like medications for […] like arthritis and those different types of, those autoimmune medications. And they may be 150,000 daltons. So it's pretty easy to reassure mom that not likely that it's gonna get into the milk because it's such a huge molecular weight. […] Some of those medications are fairly new, but we know about the chemical properties.” (BFR10)

“… just like as with any medication, just make sure that I have […] a good understanding of the classification of the medication, what the half life would be on that medication. What the absorption factor would be in terms of timeline for feeding to be able to counsel her […] of the amount of time that it would be, excreted into the breast milk.” (BFR26)

“Some drugs, especially early on, are more available to the baby because they're fat soluble, colostrum […] it takes in more of the high fat.” (BFR27)

Factors to Consider: Drug Use in Pregnancy

“But also it sounds like she's already been taking it during pregnancy so […] it's not a new exposure.” (BFR21)

“… hopefully, this has all been discussed with her already during the pregnancy, that she's on this medication, and if it's a medication during pregnancy, it most likely is going to be okay for breastfeeding.” (BFR27)

Factors to Consider: Health of the Infant

“I think it's a strategy patients don't always know they can use to lessen the amount to the baby. But I would also tell her that there are some things that they might, keep an eye on and the baby. And almost always, I warn them about sedation or irritability and poor feeding, because that's the thing that a patient would be most likely to be able to pick up on.” (BFR07)

“… parents tend to be more scared about their preemie babies and so I find like if they have a term healthy baby, I can reassure them that way but then if they have a pre-term baby, I kind of talk to them about like the other benefits…” (BFR15)

“I would look at information like if it's negatively affecting growth, infant brain development, those types of things, rather than just minor side effects.” (BFR22)

Factors to Consider: Health of the Mother

“I mean it would depend on what it is that we're treating and what the scenario to the mother's health would be. So, a good example of when we actually start a medication would be anticoagulants for postpartum DVT. So that question comes up fairly frequently, so this is not something a mother's already experiencing or already stable on.” (BFR05)

“I'll also talk about the untreated condition. So a lot of times moms in pregnancy they'll stop their medications. I'm worried about this. I stop taking it. I'm not feeling very well now. Same with breastfeeding, we'll talk about her condition and the risk for the untreated condition that specifically in breastfeeding, baby has an increased chance of having, learning difficulties, developmental delays, those different types of things with the untreated condition so that we're not just looking at the risk of the medication. We're looking at the risks of the untreated condition balancing.” (BFR10)

Factors to Consider: Information on the Drug Used in Infants

“Like if we were actually treating the baby, what kind of a dose would we use to give the baby. So, usually, the dose that the baby is getting through the breastmilk is like very small compared to if you were actually treating the baby, we'd be exposing them to much larger doses anyway...” (BFR04)

“Or we could have conversations about the mom needs to start, Keflex for a UTI. And we say, "Well, we routinely use this antibiotic for babies themselves if the baby has a UTI, so of course, breastfeeding is going to be okay." (BFR09)

Factors to Consider: Maternal Co-medications

“Oh, what other medications she might be taking, if she's on other things that might affect their brain? If she was still taking narcotics or if she takes an anxiolytic antidepressant or something like that.” (BFR01)

Factors to Consider: Maternal Dose Taken

“Depending on mom's clinical needs, if it's desperately necessary for her clinical care, and as long as her dosing is not considered on the high end. And this is where I would involve my pharmacist, […] what range is her dosing considered? Then I would advise that it would be okay to breastfeed.” (BFR14)

“I'm guessing this one can make children sleepy […] so then […] I would probably look through Lexicomp, UpToDate, to see is this safe or not. I'm guessing this is probably a dose-related thing, like how much.” (BFR17)

Factors to Consider: Risk and Benefit (Analysis)

“So, I look at the risk and benefit of the mother taking the medication. So like psych medications, for example, you don't just tell a stable mom to stop taking her psych meds so that she can breastfeed. Because that destabilizes her relationships, including her ability to bond with her infant. And so that is a huge risk to the pair, as opposed to, a relative risk to the infant being exposed to a medication.” (BFR05)

“… it's just more educating them on minimizing the amount of exposure to the baby while still getting the benefit of the medication for the mom.” (BFR26)

Factors to Consider: Select Drug Cases for Non-Resource and Resource Use

“I would start by opening my resources, since I don't have this one memorized and I would just go through them top to bottom.” (BFR09)

“Every so often we'll have a patient with autoimmune disease that might be on some stuff that I have to ask the pharmacist more specifically about the safety profile, but most of the time it's a lot of moms asking about SSRIs and on occasion pain meds, but then they fall under the Neonatal Withdrawal Symptoms category, and we usually only allow breastfeeding in the setting of that if they're in a program and they're weaning, and they're not using drugs […] for abuse as opposed to a controlled amount of methadone or buprenorphine.” (BFR14)

“… my basic drugs that I already know, most of the time my patients usually are okay to breastfeed during them.” (BFR23)

Factors to Consider: Team Approach (Present or Absent)

“And then we always have to then bring it back to the doctor and have a conversation and say, ‘Hey, like this has never been done. Here are all the potential benefits, here all the potential harms.’ And then collectively, we discuss and then make a decision together.” (BFR04)

“So then what I do is, I kind of get the pharmacy on board, the pediatricians on board, and provide the name. And then I get the information back and I say, ‘Well, I communicate with so and so, and it seems that this, this is a safe medication, I'll put it on your chart.’ And kind of leave a little note, ‘May need help postpartum with breastfeeding.’ So that the pharmacist and the pediatricians afterwards could kind of emphasize the importance of the availability.” (BFR08)

“And we, of course, being a collaborative model, consult on this type of thing. And it would be pulling in our RNs and our physician colleagues to decide if a good feeding plan and a good medical management plan for her.” (BFR26)

Factors to Consider: Time of Breastfeed Relative to Dose

“Other considerations I would have is the timing of the dose and the breastfeeding…” (BFR06)

Factors to Consider: Type of Breastfeeding (Exclusive vs Partial)

“… my concern for breastfeeding goes way down at that point once they start supplementing with anything else. Yes, and then I would ask her, of course, if she was able to fully breastfeed or if she was having to do any kind of supplementation. Because, again, that kind of partitions out, a fraction that the baby would not be exposed to.” (BFR05)

Lack of Existing Metric Use

“… not because I believe [Resource Author] ultimate conclusions…

[…]

I don't use the L1 through L5 at all, unless to notice if [Resource Author] for some reason slammed an L4, L5 on. I might take a look at why. But I don't use those codes.

[…]

I don't very often use milk plasma ratio unless it's shockingly weird.” (BFR07)

“I've heard of them, but we're not currently using them.” (BFR18)

“We've never had any education about that. We've never, like... It's never been discussed.” (BFR28)

Multiple Resource Use

“Well, yeah, so I will use the RID, but I always ask myself, okay, so there's a percentage there, but how much is the baby really getting exposed to because it will be a function of a mom's dose. So, I look at the RID but […] I take it in the context with all the other information. It's a good screening tool, but it doesn't seal the deal for me.” (BFR03)

“So I go there first, and then I go to Micromedex and see what it says. Then I go to LactMed and see what it says. And like the reason that I liked to, that I go through all of this is just 'cause every resource is gonna tell us a little bit different information.” (BFR04)

“As I work on a regular daily basis right now, I've got three different ones open […] LactMed, Reprotox doesn't have a lot, but sometimes it's useful and I have Hale's open. I've got Briggs on the bookshelf back there.” (BFR10)

“Sometimes I refer to a textbook... herbal and naturopathic textbooks if people are asking me specifically about herbs, because those are harder to find in those other resources I was mentioning. Hale's is definitely starting to expand on that, but I have some of those textbooks that I refer to sometimes.” (BFR25)

Pregnancy Categories (Using or Avoiding Them)

“I do try to avoid using any of the reproductive, like category X, category A, B, C, any of that information, with breastfeeding, 'cause it was really intended for pregnancy but it's also inadequate for discussing meds in pregnancy either. But I do see a lot of practitioners use those.” (BFR05)

“… unfortunately I still use like, the pregnancy categories, ABCD. It's just because it's a quick reference and I can say, ‘Oh, well, it's category C. Let's go back to the doctor. It's category B, okay. We're good. We're okay to dispense. Category X, we're not gonna give it to you.’” (BFR06)

“Lamotrigine is not like completely Category A, the safest drug in breastfeeding or pregnant women, but I just remember that it's considered okay […] like no serious adverse effects.” (BFR16)

Primary versus Secondary Resources

“Currently at work, we use Clinical Pharmacology. And that, that's the main resource. If there's not enough information, I will go to the package insert.” (BFR06)

“LactMed is my go-to, and then if it's unclear, sometimes I'll do UpToDate, and then Lexicomp through our [Centre Name] system.” (BFR17)

“So if I'm using Hale's book, which would be the first thing I would probably grab on my bookshelf, I would look up lamotrigine…” (BFR25)

Realities of Advising: Concern for Liability

“I'm speaking a little bit also to sort of like a liability aspect as the person giving ... Like if somebody's asking me this and I'm giving a response, there's plenty of questions that we can get asked as pharmacists that there can be like theoretical evidence for like, ‘In theory, this should be okay.’ But is it, okay, let me back up. I guess an example would be, using a dose of something that has never been studied at that dose.” (BFR04)

“Whereas, more adult providers are just scared of the liability of it getting to the baby.” (BFR15)

Realities of Advising: Concerns Relaying Evidence-based Decisions

“Now we cut and paste it into the note and the pediatrician will do what we tell them. They just will, they wanna know that we thought that we thought it through.” (BFR02)

“… and if they've had a couple of babies […] at a different site, they're gonna trust the neurologist they've been going to since age 15, more than they're gonna trust me, who now they're only gonna see for, I don't know, 15 weeks. So kind of just revamping that whole concept might take a few visits. And even at the end of it, sometimes they feel like, ‘Ah, I didn't do it the last couple times. I'm okay. I'm not gonna do it. I'm not gonna take the risk.’ So it's sometimes difficult to break those barriers if they have their mindset on that, based on someone else’s.” (BFR08)

“… who is managing these medications are always a difficult thing and how much of it is ... I think a lot of adult providers don't know about LactMed […] versus OB-GYN, I think they have an idea. But it ends up really falling on the pediatricians to have to be looking at it kind of thing.” (BFR17)

Realities of Advising: Institution Needs Resource Justification

“And in my current practice, there's not enough need for it, for me to justify that.” (BFR06)

“… we have to get all those approvals and things like that” (BFR10)

“I think for the community setting, it's really helpful to have free resources, 'cause the community pharmacy I work with is an independent, so they don't subscribe. […] The chains might have resources but the independents aren't really paying for a lot of those extra resources like that, so it's great to know what's available for free.” (BFR20)

Realities of Advising: Lack of Information About the Patients

“… sometimes they don't tell us everything about the patient or the patients, in this case, we're having a baby too. So, I think for us, the team approach especially for a pharmacist's point of view, the team approach is the best way…” (BFR06)

“In terms of prescription medications, it doesn't come up because let's say the patient picks up a medication, I'm assuming the doctor already knows or they have discussed it, and then, they wouldn't even say they're breastfeeding. So, I wouldn't know.” (BFR16)

Realities of Advising: Minimal Time for Clinical Decision Making

“I mean, I think this is great for a pharmacist who does pharmacokinetics, but I don't think this is great for a busy pediatrician in their clinic. Like they just need a yes or no most of the time, unfortunately.” (BFR01)

“People have to make these decisions in a few minutes. So if they have 30 babies to see that day, the team may be four people seeing 30 babies, 15 minutes a patient, get, it has to be pretty simple.” (BFR02)

“In the hospital […] patient room […] you may have like half an hour or 15 minutes to get back either to the doctor or a nurses.” (BFR11)

“So, often I don't have the time. I need the quick answer, and if you need to deliberate more, I fit it in in between verifying my orders, talking to physicians, rounding, helping the nurses with drug administration. So there's kind of a lot on your plate when you're in the unit.” (BFR12)

Realities of Advising: Motives of Manufacturers

“… those tertiary resources are getting their data from the manufacturers and so it's certainly not an advantage for a manufacturer to look up that information about their product. So, I can see that they spend zero resources whatsoever trying to collect or accumulate that information.” (BFR05)

“The company hasn't done the research to have in their files that says this is safe. It's just easier to say, ‘Don't breastfeed.’ And so I think it's from a liability standpoint because they haven't gone the extra mile to find out, is this a safe medication or not?” (BFR27)

Realities of Advising: Variable Patient Health Literacy

“… I do that after I've gotten a sense of the health literacy of the patient, whether that will be helpful or confusing for them. Some people really like it. Some people find it overwhelming and not helpful that they prefer the summary version that I'm giving them.” (BFR07)

“I think just telling them a little bit more, 'cause all of our counseling needs to be at a sixth grade level, unless you know that the knowledge of the parent is advanced, and then of course you know you can get a little more sophisticated in your explanation.” (BFR12)

Refer to Other Provider

“Because the baby is so young, I would also have extra considerations, and probably refer back to the doctor. A lot of the resources do not go that young for me.” (BFR06)

“I would maybe just share some information with the patient and advise them to contact their provider.” (BFR20)

“I would either look at LactMed or I would ask one of the providers to come answer the question for them.” (BFR28)

Reliance on Other Provider or Resource

“And really, mostly what we do is consult our lactation consultants. So, from the nursing perspective here, that's our first go-to… is accessing our lactation consultant team and then our physician team.” (BFR13)

“We have a designated NICU pharmacist who is very readily available to us. In fact, we sit in the same writing area. We are typically in communication if we're running TNAs or TPNs for the babies. But questions about mom medications, [I] frequently […] go to them for a lot of the ones that are not as straightforward.” (BFR14)

“And then, I will call our pharmacist if we're, especially if it's a combination of medications and we're not too sure about are usually where we go for information.” (BFR18)

“… then if the information that I'm getting is not making me feel comfortable, then I also have the resource at [Centre Name]. I just call our lactation office at [Centre Name], and then I talk to one of them, and I have a couple lactation consultants…” (BFR23)

“I feel like it's a quick way to kind of assess if something... I mean, if something is an L5, to be honest, I don't really dive much deeper to see why [Resource Author] came up with an L5. I've been using that […] rating system for long enough that I feel like if it's an L5... I'm done.” (BFR25)

Use Combination of Experiences and Resources

“The focus has to be on not just saying, ‘Okay, I looked it up in Hale's and he says, no, we're not gonna allow it,’ but to sit back and think, okay, wait a minute. What's the science about how much actually gets in? Is there an option to try another medication? No. Could we give the baby some of her milk?” (BFR02)

“I'm a little bit familiar with this medication. I know that larger amounts gets into the milk can increase the risk for sedation, but I would take the time with some of these medications that I do know are more concerning.

[…]

But then […] take a look at the half life and kind of understand the medication that those can be sedating and because of the half life, they can build up and cause that sedation. So, you have to be able to not only look at the Relative Infant Dose, but consider it within the greater context.” (BFR10)

“I know, previously the old literature stated that you should not take SSRIs while pregnant 'cause it increases risk of persistent pulmonary hypertension in the newborn. However, I think the new guidance says that mom should be more stable and be taking their SSRIs while pregnant.” (BFR22)

Use of Package Inserts

“… if some other question that [needs] more details, we can go into look at the package insert in each bottle if needed…” (BFR11)

Use of RID in Specific Cases: Comparing within Drug Class

“I might use the metric in comparing between drugs of the same class.” (BFR05)

Use of RID in Specific Cases: Explain a Range of Outcomes in Infants

“I might give the range and the relative infant dose. I might say that there has been a general rule that […] medications used during the breastfeeding, maybe less than 10% of the mom’s weighted dose. And with this one you can see a range, and so that's why some persons might have more caution, but here's what we see when actually moms have breastfed with the medication.” (BFR21)

Use of RID in Specific Cases: Mother on Co-Medications

“… if I see that it's got a higher relative infant dose and let's say it's a woman who's on multiple medications and I'm a little concerned…” (BFR02)

Use of RID in Specific Cases: Mother with Conditions

“I'll take a look at the RID and milk/plasma ratios when we're talking. Occasionally I'll write a consult in the chart. For example, […] there was a mom a couple of years ago […] she had some comorbidities and […] the physician out in East County did prescribe codeine, and that's when we were not doing that, because we weren't looking at polymorphisms in the mom and the baby.” (BFR12)

Use of RID in Specific Cases: New Medication

“And so that's where I would use those metrics then of what's getting into breast milk, or what the infant's relative dose would be, is in a case like that where I'm choosing between drug classes for a specific new indication.” (BFR05)

Use of RID in Specific Cases: Reassurance Along with Other Resources

“And most medications have RIDs that are single digit percentages or even decimal point digit percentages. And you look at this in a really quick glance and you say, ‘Ah, it's probably okay.’ […] But the, the RID is your first point of reassurance for most medications, except for the few where the RID is actually higher than 10%. Which in my line of work, [of] the medications that I get asked about most often, they're few and far between.” (BFR09)

**Disadvantages of Existing Resources**

Areas of Subjectivity

“… it uses those codes. And, it's opinionated. It's gotten better about that. But it can be, this happened to two patients and I now think blah, blah, blah. I mean, breastfeeding research is a victim of small subjects and […] I'm like we would never, never accept it […] but that sometimes that's what you've got.” (BFR07)

“From a layperson's standpoint who is out there Googling, ‘Is it okay for me to take narco while I'm breastfeeding,’ they're gonna come across an ocean of stuff. And much of it opinion, much of it based on one cherry-picked, selected study or another and pulling them towards whatever conclusion it is that they're probably most likely to dive towards in the first place.” (BFR09)

Co-medications Not Considered

“So if mom's taking three medications, how can I look up if that's more concerning or not? So I just have to in my head, oh, these all three affected essential nervous systems and I have more concern would be the biggest ones. (BFR01)

Easily Outdated

“We don't really use any books anymore, just 'cause they get outdated.” (BFR13)

“… but the problem is if you don't buy a new book every single year, who knows what information is changing as well. So that's the negative about the books. Although, I had every drug possible that you can think of in those.” (BFR23)

Effect on Milk Not Considered

“… the issue about, does it affect milk supply? I feel like that comes up a fair bit, so I dislike that Hale doesn't have that.” (BFR01)

Inaccessible

“I know there's like different, there's books, too, that we have in like, especially in our newborn office. I just haven't used them as much since they cost money.” (BFR15)

Infant Age Not Considered

“… there's not great, like specifics towards preemies. And most of the time there's not that consideration at all and they have a different renal clearance for sure. They also have a definitely different weight.” (BFR15)

“Or the infant's age is not taken into the consideration at all.” (BFR16)

Maternal Dose Not Considered

“But there's no like dosing information or anything, I will say that. This is just like general overview.” (BFR28)

Non-average Cases Not Considered

“I would say that […] it doesn't elaborate enough on, the upper and lower curve of… issues that might be coming, or other complications that might come. It just gives us the mean or medium of the information out there. And so, unfortunately not all babies fall in that middle of the bell curve.” (BFR06)

Overreliance on a Single Resource

“… there's a ton I don't use like, the […] package inserts are so problematic and that's what families sometimes have access to where it doesn't give any information about breastfeeding or just says, "Talk to your doctor." Or it says shouldn't breastfeed based on no issue. They just don't want the medical legal liability.” (BFR01)

“… even in RID, you're clinging too much to a proxy and not like looking through all the information. So, the disadvantage is you really have to look through all the information being presented and come up with a plan and apply it to your patient.” (BFR03)

“Relative infant dose? Yes, yes. Although, I think people use this 10% cutoff for relative infant dose, and it's not true. I saw somebody do that recently. And I thought, "What are they talking about?" If it's something toxic, then it's a little baby, yeah, you care, even if it's 3% or 4%, or whatever. And if it's not something you're very worried about, okay, like something like that right now, and it's 25%, but you could give it directly to the baby, well, then you might not care.” (BFR07)

Overreliance on Case Reports and Published Data

“I think the disadvantages in the literature that [Resource Author] uses to draw from it. So, again, I don't think there's really any modeling going on. It's just, what is this study shown? It's often two samples here and five samples there, but that, that's what we have. So something where there's some more scientific modeling would be fantastic…” (BFR02)

“I guess in reality is that a lot of the times when I go to LactMed, most of the medications will say ‘Insufficient Data to Advise Against or For.’” (BFR04)

“I think the main disadvantage to everything is that because there's no human studies, we don't know exactly what the possible side effects and risks are.” (BFR24)

“I feel like a lot of that hasn't really been studied well. So that, that's the problem, right? Like we don't do a lot of studies.” (BFR28)

Perceived Lack of Reported Information Due to a Resource

“And then there are times where I'm like, "Ah, LactMed doesn't have it," […] it's not quite the prescribed medications, it's the supplements that I have a lot of issues with.” (BFR17)

“So, if you use their resource, as I have for a long time, that it's got some problems. And one of them is that they don't include the different formulations, even though there, it say Wellbutrin, bupropion. Most patients do not use the 150, just plain old non-extend. They use a 300 extended release. And so, the data in there for it is not actually correct for that particular [case].” (BFR07)

Too Broad

“Other tertiary sources, like Micromedex and Clinical Pharmacology are easy quick grabs, but they tend to be a little pale in what quantity they provide in information.” (BFR05)

“I think a lot of them just leave it up in the air. Per provider discretion is something I see across a lot of resources, and so that's when I end up having conversations with the pharmacist reading up case reports” (BFR14)

“It's not gonna go into kinda more the bioavailability of what's getting to the infant or the clearance. It doesn't have like those further steps of recommendations and so that is a disadvantage.” (BFR15)

“Lexicomp, for example, […] it does not have that much information about breastfeeding. And, infant exposure, it usually just has a little blurb or one sentence, so it's not very complete, or I cannot depend on that single resource. It's not all-inclusive. So if I would not use Lexicomp alone, it would just be a starting place, just to see maybe where I should branch off to look up for more information.” (BFR22)

Too Much Information or Text-heavy

“Disadvantages, sometimes they can be slow to get to the point. The more exhaustive listing of all the potential issues that have been studied can still be a little bit off-putting and can, lead you to be anxious about making a decision that ultimately is the best one for your patient.” (BFR09)

“… when I'm looking at Hale's, it's very easy to see that table. With LactMed because it's presented in a narrative structure, I have to read through that. I can still find that Relative Infant Dose, but it's harder to dig that out while I'm talking to someone on the phone.” (BFR10)

Unclear Conclusions

“I think UpToDate and Micromedex, […] they don't always give […] a clean summary statement or like a final, "At the end of the day, this is what is recommended."” (BFR04)

**Advantages of Existing Resources**

Accessible Through the Institution

“And since that is a database readily available at my institution that my institution pays for anyway, I will sometimes go there first…” (BFR03)

“… our computer system link directly to ClinPharm […] so I just click into it. This may take about five seconds so that's a number one I'm going to before Google and […] ClinPharm doesn't give me the answer first.” (BFR11)

Comprehensive

“Someone wanted to know if they could use monk fruit and breastfeed. And Reprotox is the only one that even have a monograph on it and just said nothing is known.” (BFR07)

“… at least that's very helpful, like, 'cause they have gone through everything.” (BFR17)

“So they have everything in there that I feel I need” (BFR21)

Distinguishes and Provides Various Types of Data

“… but then also goes through and breaks down the information as to whether or not the data's coming out of animal studies, or human studies, or case reports, or if there is measured maternal milk levels or relative infant doses.” (BFR05)

“I also like the fact that it describes both the information on the exposure, the maternal side and the infant, so it separates it out in terms of if […] there's any data on levels and infants, if there's any data on infant response and adverse effects and whatever.” (BFR07)

“I do like to be able to see […] the Relative Infant Dose, some of the narrative information. For example, some of those medications we know are given directly to pediatric patients and that will be in the narrative. And that will give us a clue that yes, if it's given to a pediatric patient at birth, acyclovir, not likely to be an issue, regardless of all of those other things. So, there are a lot of those different pieces of data and narrative that help to build that risk statement that's in those different databases.” (BFR10)

Evidence to Support Use

“It works sometimes because they have gotten a lot better over the years and there's some data to back that up, at least one study I know of, showing their improvement over the last 20 or so years.” (BFR03)

“Like it would need to be validated and I, as the clinician, we need to understand where that information came from so that I trust the source.” (BFR05)

Familiarity

“Purely for, probably mostly for convenience and familiarity. So, I think the way, when I was in my training, LactMed was sort of considered the gold standard.” (BFR04)

“It's what I'm familiar with when making clinical decisions in infants, with even just medications being given to them.” (BFR22)

Generally Accessible

“It's readily available on any computer that I'm sitting next to. I don't have to log into anything since LactMed is free.” (BFR01)

“I use the app on my phone usually, and it's free. So even in the community setting where they don't have any […] institutional resources…” (BFR20)

Patient-friendly

“… and then the MotherToBaby, the advantages, I can give it to families with lower education level and they can use it. I guess that's not beneficial in terms of my making a recommendation, but it's beneficial and, and giving that information to the family. I can copy it and […] we'll have to put it on our discharge summary so we can communicate the information to the outpatient pediatrician, why we made a decision.” (BFR01)

Summarizes and References Evidence

“I think it's more […] pre-digested. The information, the research is kind of, it's almost like a meta analysis or someone else has kind of like, looked at is, or, and also […] it's concise.” (BFR19)

“I guess my thought would be that it's laid out that there, for example, LactMed will talk about any studies that have been done […] will look at general safety profiles…” (BFR24)

Summary Statements

“We have our kind of summary on there, for example, we'll have a summary based on the available information, there's no conclusive evidence, that type of thing. And then we'll have, we'll pull from some of those other databases like LactMed. I'll pull the summary statement and put that in our database so that as we pop those up, we can see some of those other databases compared.” (BFR10)

“A couple sentences, it's really easy to get to, and also […] understandable language.” (BFR19)

“I mean in LactMed it's pretty specific… you don't have to read too much to get the bottom line information. So I do like that about LactMed…” (BFR23)

Trusted Authors

“And they've brought on a bunch of editors who have skill sets in those patient populations, including lactation. And so, that's the other improvement. Just their editorial board.” (BFR03)

“I think LactMed also has the best description of the studies that they've mentioned. Probably 'cause it's written by pharmacists.” (BFR04)

“… like with InfantRisk, I feel like that I trust as a resource because there are several pharmacists who work for... It's through the [University Name] and so the whole department is pretty much kinda looking into this and they are the ones going through all the studies, so that I don't have to kind of thing.” (BFR25)

“… there's someone that's gone before and it's a standardization that you can lean in on.” (BFR26)

Up to Date

“The advantage of LactMed is just that it's updated so frequently…

[…]

So I always feel like it's very well updated. You can see the date that it was updated.” (BFR02)

“Yeah, it is nowadays, I don't use textbook, partly because we cover so many sites, and these books are so easily outdated that we don't go to books anymore. So these are the online resources I use.” (BFR08)

**Advantages of the UAR**

Addresses Clearance Differences

“Okay, here's the dose in the milk then what's the bioavailability to the baby, what's the baby's clearance ability from their bloodstream? […] How much is gonna kinda stay around in the baby? So it's just a better in some cases I would use that information and be like, ‘Look, baby's gonna clear at the area under the curve like this ratio it's staying pretty low for 95% of babies.’ […] so having more information is always appreciated and […] helpful.” (BFR15)

“… didn't it look like the UAR does like metabolism too? […] Like if it's slower and that's an area 'cause I don't know that what the person's are they fast or slow? That's helpful to have factored in.” (BFR21)

Addresses Exposures (AUC)

“But this just gives you an even more exposure assumption than the RID does. So it's better.” (BFR03)

“… it probably would be accurate in assessing what the risk was to the infant based on maternal plasma levels, so I would think that the value itself would probably be very accurate in terms of infant risk and that would be an advantage.” (BFR25)

Addresses Multiple Considerations

“I like that it provides another metric, another input, that appears to be relevant and take a lot of factors into consideration and present them quickly. […] I like that, if it were added to my existing resources, it wouldn't be adding a paragraph. It would be adding a number. I like that. I see it as a positive.” (BFR09)

“I do like that you guys take […] into account the clearance, […] especially with the renal and hepatic clearances and metabolites, too.” (BFR14)

“… it helps to kind of take into account all the different factors, such as the age and the clearance, like renal clearance and, like our preemies have much less renal clearance than older kids.

[…]

So, and taking into account more than just, like, just the amount, like the dose in the milk, and then, like you're saying, so we're talking about, […] kinda all those steps.” (BFR15)

“… I like how it incorporates more aspects of, of the situation, where often […] I'm not relying on individual metrics except in a very broad approach or if it's something like biologic and oral bioavailability, or often the relative infant dose is simply […] this cutoff and here look it matches, and here oops it doesn't, but what do we know for other moms who have decided to breastfeed.

[…]

the UAR that you'd be getting more specific to the situation.” (BFR21)

Addresses Scarcity of Published Information

“… to me looking at it, I think […] it's more data-driven. […] I mean, the majority of medications, we don't have studies on. So you're going by case studies or reporting or whatnot. So I feel like this has much more data-driven, sort of evidence to back it up, and I like that.” (BFR18)

Addresses the Age of the Infant

“… the UAR, I like that it is more specific to the, yeah age of the baby and the individual, […] because […] the way I counsel is more of broad strokes.” (BFR21)

“I think if we're really able to look at the dosing or what we think would be the probable dose, especially for those early days from like, the 0 to 14 days or the first two weeks of life where babes can be most vulnerable, I think that it would be interesting to get an idea of what we think, the concentrations are and then to really be able to talk to our patients with a better educated guess.” (BFR24)

Addresses the Maternal-infant Pair

“I mean, it's like everything in mother-baby, there's two patients involved. It's not just one patient involved. It's how much the mother takes, what gets into her milk, how much the baby takes in, what the baby's biology is. So this is actually taking that and putting them together, which is really nice because so many times it's really just what gets into the milk and it makes people worry and not think even about the oral absorption.” (BFR02)

Addresses the Worst Case Scenario

“Well, the main advantage is that it gives you almost like a worst-case scenario because you're comparing the 95th percentile to the median.” (BFR03)

“And this one seems to add additional factors that might explain that it actually is even safer still. Although maybe I'm wrong. Maybe there would be a scenario where there is currently a medication that existing resources say is most likely safe, but this new number shows a point of risk, where we might want to avoid it. I mean, I suppose that's possible, and that would be interesting.” (BFR09)

Can Share with Other Providers and Patients

“Actually, it's easier to tell patient, ‘The number you look for is 0.7, but this drug has 0.2.’ So yes, it's much, much, much lower. Kind of like a radiation dose.” (BFR08)

“If something's more complicated, I could definitely see where this novel metric would be useful to help […] share with a neonatologist and talk to the mom's primary physician to have that discussion and document it.” (BFR12)

Numerical Metric and Objective

“In the NICU we're very number focused, so I think it definitely helps to put a metric and a number to things.” (BFR14)

“I like that it gives you a value, like if you were educated how to interpret that value […] correctly. It's, that definitely is a lot simpler.” (BFR28)

Opens up the Thought Process

“But this would kind of reinforce that […] this is only in the 95th percentile. So, this isn't going to happen with every patient. But, you don't know […] if your patient is in the 95th percentile or not, so you have to plan for this. And so there needs to be kind of a monitoring plan for the mother and the baby and the physician. And if we're not able to do that maybe you should consider supplementing with breast milk to reduce the exposure.” (BFR03)

“I mean the histograms are nice to look at the relative risks, but relative risk is relative risk. Some people are adverse to any risk. […] So I think it's still important to let her know that there is a risk and […] to talk to her neurologist and unless her seizure control isn't great on lamotrigine and then it would be definitely worthwhile for her to try another agent. But if she's got good seizure control and hasn't had a seizure in a while, I mean definitely now is not the time to change that.” (BFR12)

“So I would look at the age of infant, the dosage, and then be able to better […] look in the boxes, percentile, and then find a UAR and help counsel her about the safety of it.

[…]

Whereas, given current resources, what we would do is just look at the medication itself and whether it's safe or not. So, it's more of a yes no, and this gives more of an in-depth, and personalized look at each patient scenario.” (BFR13)

Understand Existing Observations, Evidence, and Recommendations

“… it's reassuring that the infant relative exposure is low, […] I think this is really interesting. I mean, we think of baby's renal function as... borderline. They only have 10% of their glomeruli when they're born and then it gets better and better. So I'm kind of surprised to see that peak and then dropping back down, which I don’t understand.” (BFR02)

“… I think that the biggest advantage I see in it is that relative comparison to maternal dosing. To use that as a graphical representation that I can show a patient and give them reassurance. I wouldn't anticipate that your general level patient would be able to interpret data out of a graph like that, but just to be able to visually say, ‘Okay, here's you and here's baby and see how these bars completely don't touch each other.’ […] The escitalopram graph is a very good one.” (BFR05)

Visual Representation

“… I am a very visual person, so I think that it's really great for visual people and it shows break points in ages…” (BFR12)

“I just saw that UAR is green all down the line, which is wonderful. But I think the interpretation, like that bottom plot, is probably the most helpful part of it all.” (BFR17)

“So, I liked [that] you showed me the math where it says upper AUC ratio, and it shows me what the numerator and denominator is, and […] I understood it even better when I saw the diagrams with the dotted lines. I think that just described it really well to me 'cause I'm a very visual person.” (BFR22)

**Disadvantages of the UAR**

Co-medications Not Apparent

“… it doesn't combine meds and it doesn't seem like this one does either.” (BFR01)

Difficult to Understand or Too Complex

“… it's a little complex, the way this is, nobody would be able to look at this for each drug and make an assessment. You would need to put it together for us.” (BFR02)

“… it just adds more metric, to a decision making process that I wouldn't necessarily share all of that with a patient as it can be kind of overwhelming or too much information.” (BFR05)

“I think it's pretty hard to analyze unless you're really super like, into the research part of drugs, which most doctors aren't.

[…]

But not looking at these numbers. We need it more digested, we need simpler.” (BFR19)

In utero Exposures Not Apparent

“… this doesn't account for what they already were born with. That case she was born with, right? This is assuming a birth blood level AUC of 0 in the infant.” (BFR03)

Lack of Maternal Perspective Consideration

“I think that your metric kind of just looks from the infant perspective. I don't think that it necessarily weighs in anything about the maternal perspective.” (BFR05)

Limited Information on Adverse Effects (Exposure-Response Relationship)

“… unless there's any kind of clinical coloration to that, the number isn't terrible meaningful to me. It could be a very high number, but if it's no clinical effect, then that wouldn't matter. It could be a very low number, but if it's a particularly toxic medication like an oral chemotherapy or something, then that would mean something more to me, relatively at least.” (BFR05)

“… drug doesn't [affect] the baby, then no risk, […] even though the high concentration is… and but it doesn't cause […] risk to the baby, it's still okay, you know?” (BFR11)

“I think the question still is what does it do for baby? Like do we know if there's any adverse effects in the child in a developing brain of this small amount of exposure of lamotrigine or escitalopram or anything and we, most of the time we don't know.

[…]

Yeah, that would be interesting like if they're up at the higher levels, what is that doing if it's able to be therapeutic for the adult, […] and then, I think taking into account like what, what we know about how it affects adults and what are the adverse effects in adults to is some useful information. Like if you know a medication causes elevated transaminases in adults, it's not like as one of their side effects or you're watching like platelet or neutrophil counts in adults because of this medication, then that's probably not great for babies if it's reaching a therapeutic level, then that's not good.” (BFR15)

Metabolites Not Apparent

“Well, we didn't talk about metabolites yet. I mean, would there be ... Are you thinking of combining the two drugs in the one giant AUC, one combined AUC? When I say two drugs, I mean the drug and its metabolites” (BFR03)

Multiple Administrations to the Mother Not Apparent

“… at least in just the one single example that you gave me, it was just a one-time dose. This mom probably has been taking it, or will be taking it for a long time, so I don't know how that's going to change the total exposure to the infant.” (BFR22)

Path to Understanding the UAR: Exposure Comparisons

“So we're looking at the difference between the 95th percentile and mom's median, and the wider that's staying from the lower the exposure, right?” (BFR14)

Path to Understanding the UAR: Interpreting the Exposure Estimates

“And so the closer the dash line, the higher the UAR, and the higher exposure to the infant. […] Yeah, you have higher and lower, but what's safe and not safe is what I think I'm having the hardest.” (BFR18)

Path to Understanding the UAR: Interpreting the UAR

“I don't know what the acceptable UAR is. That's the part that I didn't understand. What is the ratio supposed to be?” (BFR08)

“I can see a UAR of 0.24 at 0 to 7 days, and then it goes up to, up 0.44 at 14 to 30 days, but […] that doesn't mean anything to me. I mean, I know it increases, but […] relative to how that affects the baby doesn't mean... I don't know how to interpret it.” (BFR18)

Potential to Appear Subjective or Misinterpreted

“I can see how people might look at that and say delay breastfeeding until it drops. But […] that impractical. If you see that curve that goes over and say, “Okay, we'll just wait till this point as some kind of arbitrary safe point.” I wouldn't assess it that way, but I'd be worried that some people might look at that and interpret it in that way.” (BFR05)

“… there's always an issue when you account for all those things for them. And then, they also account for, again, on top of it. So, I guess that's the question. […] I think most of them know to be careful with newborns. So, if UAR is taking that into account, if they take into account, again, that is a newborn, is that going to make it look riskier, for example?” (BFR07)

Prematurity Not Apparent

“And then what about prematurity? Like, is this just chronologic age? Has this taken to gestational age or is that part of the vulnerable piece? Because I think there's a lot in the NICU trying to look at, ‘Is it okay for this baby to have its mother's milk or not?’” (BFR01)

Unusable in Current Form (Too Novel)

“I think it would've, if you gave me some guidelines of how to interpret the ratio. But right now I'm still lost.” (BFR08)

“The biggest disadvantage is that those different teratogen information specialists around the world just aren't, haven't used it before. So it's a novel approach.” (BFR10)

“But for me specifically, because I'm not familiar with […] it doesn't mean as much to me, honestly.” (BFR25)

**Strategies to Improve the UAR**

Add a Summary Statement

“And if you can find a way to translate this into a statement, typical mother, typical baby, somehow to make it practical.

[…]

whereas LactMed is a good balance of putting the data together and then giving you a common sense recommendation. It's really, that's part of what makes it so valuable. So trying to do something like that with this.” (BFR02)

“If you gave me the information in a form like this that was quick to take in that I'd already been trained on, if I was already familiar with this new number, this new metric, and I've already had training on it and already been convinced that, yes, this is a reliable metric, and you can present it to me in a short bullet point format, such as the RID that I already have access to in the emergency department, yes, I would absolutely look at it every time.” (BFR09)

“… so if I was reading it like online or wherever, I would have like a statement of like this as kind of a conclusive statement to make sure that it's being interpreted correctly.” (BFR15)

“I like summaries. So, like, […] ‘This means this, this high number means this, this low number means this, this is what it means, okay, this is how we got to this point.’” (BFR23)

Combine the UAR with Another Resource

“So this alone doesn't help me decide, but in parallel with the other things that I would see in a LactMed reference, which would gimme a little more information, I would feel comfortable.

[…]

It depends how you're presenting it. So if you're melding it, let's say into LactMed or something. They trust LactMed and they're, and you don't have to tell them all that. You just have to tell them what they wanna know.” (BFR02)

“… my question is […] when these metric develop, can they […] incorporate into ClinPharm or other […] resources?” (BFR11)

Explain More About How the Model Was Made (Inputs and Assessments)

“… when you report the UAR, have it be available to know like where that, where that data came from. So like I was saying, in how many breastmilk samples from how many patients. And then with the adult AUC data, like what is this AUC represent? What dosing ranges is this AUC representative of? Like, […] we use like crazy doses that are not FDA approved. […] So I don't expect, a group like this to go and test these off label doses that we use 'cause that's you know, that's not normal. But, that could help me know whether or not I can apply the UAR to my patient better.

[…]

And, I feel reassured that you are validating with actual infant samples as well.” (BFR04)

“So it's a novel approach. It would take some training to get used to understanding all of the different pieces that are in that model and how to interpret it.” (BFR10)

Explain More About Its Advantages

“... most people aren't gonna understand, but if they know that it takes that into account, whereas these things don't, you'll be like, ‘Oh, okay. That's great.’” (BFR01)

“to discuss the benefits that it gives more information about a drug, and based on baby's age. So I think if everybody understood it, and saw that it definitely gave them more information, it would be very usable.” (BFR27)

Give Specific Training

“… disseminate the information, having practitioners get accustomed to it would be big. […] I honestly, I think this would be a one lecture or a one presentation type of topic to discuss to people. And I think, a lot of practitioners are smart enough to understand, so.” (BFR06)

“Maybe it would be much easier if, if I sat through a talk and understood for different medications, what it meant.” (BFR08)

“So, I think that, to be completely honest, maybe because my, like I graduated [many] years ago, I'm not used to looking at these things anymore. So, maybe some people might need some kind of, maybe just like 10 minutes of training to completely understand it.” (BFR16)

“Like, breastfeeding is not talked about or thought about really in medical school at all. And so the question is can you influence pharmacological kinda things, or at least introduce the concept of […] how do you interpret these things and think about people as a whole? I mean, I think all of medical education is going towards more of a wholesome view of medical education, so I think this is sort of the time to, like, start introducing it, as well.” (BFR17)

Make the Metric and Path to Its Use Audience-dependent: User Friendly for Non-Pharmacists

“I think this is great for a pharmacist who does pharmacokinetics, but I don't think this is great for a busy pediatrician in their clinic. Like they just need a yes or no most of the time, unfortunately.” (BFR01)

“Whose knowledge of pharmacology, mine included, is dated and minimal really. And we're more likely to call somebody up and ask them, call up the pharmacist and ask them what we should do. But I think understanding how the model was built and having a statement about that. 'Cause if you show them this, they're not gonna take the time, but if they know that it's a model built on X, Y, and Z, that would be another summary statement. And then this is what we found with this drug for this age, baby or whatever. So it's really how you present the educational part of it's gonna be really important.” (BFR02)

“but I also think just to help clinicians get it because this is a harder topic […] explaining that to them in a accessible way... would be one of your challenges.” (BFR25)

Make the Metric and Path to Its Use Audience-dependent: User Friendly for Pharmacists

“I think it's complex enough, it needs like I think this is probably great for like a pharmacist or somebody who's good with these sorts of ideas.” (BFR01)

“I think so. I think in pharmacy school, we're taught basic statistics, we're taught about AUC. I think […] this document is very clear and to the point, and I think pretty much any pharmacist could understand this.” (BFR22)

Make Visual Representation Essential

“I think having a picture is always helpful for people, especially, that don't read well. So if you could show something to the family... And I was thinking it might be nice like the mom could color the whole thing and then the baby could move at the different ages 'cause then they have a better idea. Like this could be a light blue and this could be a dark blue. And then it shows where they overlap. Like I think that helps people too is to kind of see that.” (BFR01)

“Yeah, I think the table is probably the most helpful, especially […] the providers are much more familiar with the box plots and things, but the bedside nurses and the lactation consultants probably aren't. So from a nursing standard the table is probably the most user friendly.” (BFR13)

Overcome Simulation Skepticism

“I think that piece is difficult for people to understand too, right? This simulated idea, like what does that mean? Sometimes people don't trust things as much if it's like from a computer. So helping people to understand that.” (BFR01)

“But, PBPK modeling has been around a long time and not a lot of clinicians know about it. But when a drug company wants to study drug and children, which they're required to do now in the US, probably in Canada, too, when the drug company brings a drug to market, they're required to study it in children and the first step is to do some PBPK modeling to see, okay, what do we think the right dose will be? And so, we're now just applying that same concept to how much, what do we think the dose is in the breast milk, and […] this might make people more excited about it because this is just the normal way in which we figure out what the dose is.

[…]

we do it by this modeling […] which is a lot more precise. So that, that can be […] eye-opening to people. […] Every drug you prescribe to a child, it probably was studied in this way originally. Now we're just bringing that to drugs and lactation. It makes a lot more sense […] something that's normally done in pediatric drug development. […] It's something you have been applying in clinical practice and just didn't realize it.” (BFR03)

Provide a Definitive Bottom Line

“So, I mean, I mean gonna reference back to an old outdated system that we really don't use anymore, with like pregnancy categories, how we had the ABCDX, and what those numbers mean. So, if there's a way for us to say like, a UAR of 0.5 is on a yellow range like, it's okay, or cautious. Whereas, a UAR below 0.25 is green, or something like that. […] While this number is below in this range, let's go.” (BFR06)

“I could foresee myself really appreciating of the metrics that this rate of UARs means that there is a X percent probability that the baby could have X outcome. […] That means that baby will have QT prolongation. Or, if you accept a UAR of a […] of a higher value, then that percentage of that risk decreases. So it's almost like a probability type of scale, because at the end of the day clinicians are going to care more about that than the actual milligrams of drug circulating in the baby, because what does that even mean at the end of the day if, that X milligrams circulating in the baby doesn't end up hurting them?” (BFR14)

Provide a Greater Maternal Emphasis

“I think that'd be significantly helpful if it included the mother. […] I feel like women are sacrificed for reproduction a little too much and […] I appreciate the concern for risk to a newborn infant […] and we certainly have an instinct to protect them, but also, it shouldn't be at the risk of [the mother’s] health. And so I would want something that kind of emphasized, taking good care of the mother so the mother can take good care of the infant.” (BFR05)

Provide Guidance to Interpret the UAR Metric

“We would have to know how to interpret the numbers, and not just be given a number and then we just shrug our shoulders at it.” (BFR14)

“But if you come up with some sort of guidelines. And maybe the guidelines are not general. Maybe you're not like, ‘A UAR of less than 0.3 is okay’…” (BFR20)

“So if something like, in general, if your UAR for this medication is less than 0.3 […] we're interpreting this for term infants from age, 0 to whatever, we feel that this is safe. I would like a better range in terms of […] these are acceptable levels...” (BFR24)

Provide Prospective Predictive Evidence

“I guess missing any prospective data that tells you which choice is the best. […] That's the next step, right? I mean, that's where we go from here is to test these prospectively to see if they're predictive. But I don't know if I would call that a disadvantage, because no one has that currently. There is no metric that currently gives us that option.” (BFR03)

“Buy-in in my department consists of showing that, if you use this metric and base your decisions on it, that patient outcomes are going to be as good or better. So in this case, do a study that shows […] if we applied this metric to breastfeeding decisions for 10,000 infants, as opposed to the current metrics, these would've been the outcomes. This would've been what you recommended, and they all would've been just as good or potentially better off.” (BFR09)

“But at the end of the day, though, this metric would still have to undergo rigorous clinical application and study to see what these values actually mean and […] what it means in the baby clinically. Because at the end of the day, a number's a number, but the baby's clinical outcome is going to be what's most important.” (BFR14)

Separate by Specific Cases and Scenarios

“It would be tremendously helpful if you took, for example, several different psychiatric drugs or several different anticonvulsants. Or, I learned thus by comparison and contrast, and I think that over the years, I mean, that is what my many years of experience provide...” (BFR07)

“Yes. That's helpful for the specific drug. It would be helpful though to compare some of the different drugs that I'm more familiar with to see how that works and how that process happens.

[…]

it would be helpful to have a variety of different medications with different models. So I don't want see five medications and all of the models look exactly the same. I want to see 10 different medications and every model looks different. And then a description of why that model looks different for this medication compared to this medication so that we can start to understand how to interpret the differences in that model.” (BFR10)

“Yeah, I definitely could see it being used in the NICU setting. I think you would have to like break it up by gestational age, too.” (BFR15)

**
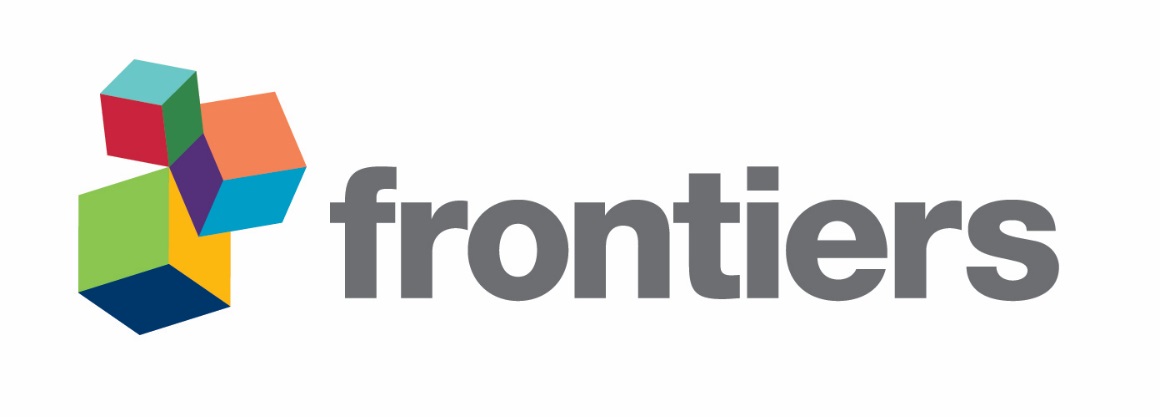
**
